# Survival in Patients with Colorectal Cancer and Isolated Brain Metastases: Temporal Trends and Prognostic Factors from the National Cancer Database (2010–2020)

**DOI:** 10.3390/cancers17152531

**Published:** 2025-07-31

**Authors:** Zouina Sarfraz, Diya Jayram, Ahmad Ozair, Lydia Hodgson, Shreyas Bellur, Arun Maharaj, Vyshak A. Venur, Sarbajit Mukherjee, Manmeet S. Ahluwalia

**Affiliations:** 1 Miami Cancer Institute, Baptist Health South Florida, Miami, FL 33176, USA; zouina.sarfraz@baptisthealth.net (Z.S.); diya.jayram@baptisthealth.net (D.J.); sarbajit.mukherjee@baptisthealth.net (S.M.); 2 Department of Neurology and Neurosurgery, Montreal Neurological Institute and Hospital, McGill University, Montreal, QC H3A 2B4, Canada; 3 Division of Medical Oncology, Department of Medicine, University of Washington, Seattle Cancer Care Alliance and Fred Hutchinson Cancer Research Center, Seattle, WA 98195, USA; 4 Department of Translational Medicine, Herbert Wertheim College of Medicine, Florida International University, Miami, FL 33199, USA

**Keywords:** colorectal cancer, brain metastases, stereotactic radiosurgery, systemic therapy, prognostic factors, healthcare inequities, overall survival, treatment modalities

## Abstract

**Background:** The development of brain metastases (BM) is a relatively uncommon but significantly adverse event in the spread of colorectal cancer (CRC). Although management of CRC BM has improved with advances in imaging and systemic therapies, clinical outcomes remain poor. **Methods:** This retrospective cohort study used the U.S. National Cancer Database to evaluate survival outcomes, treatment patterns, and prognostic factors in CRC patients diagnosed with BM between 2010 and 2020. Patients with isolated brain-only metastases formed the primary analytic cohort, while those with additional extracranial metastases were included for descriptive comparison. Multivariable Cox proportional hazards and logistic regression models were used to assess factors associated with of survival. Proportional hazards assumptions were tested using Schoenfeld residuals. Accelerated failure time models were also employed. **Results:** From a cohort of 1,040,877 individuals with CRC, 795 had metastatic disease present along with relevant data, of which 296 had isolated BM. Median overall survival (mOS) in BM-only metastatic disease group was 7.82 months (95% CI: 5.82–9.66). The longest survival was observed among patients treated with stereotactic radiosurgery combined with systemic therapy (SRS+Sys), with a median OS of 23.26 months (95% CI: 17.51–41.95) and a 3-year survival rate of 35.8%. In adjusted Cox models, SRS, systemic therapy, and definitive surgery of the primary site were each independently associated with reduced hazard of death. Rectal cancer patients had longer survival than those with colon primaries (mOS: 10.35 vs. 6.08 months). Age, comorbidity burden, and insurance status were not associated with survival in adjusted analyses. **Conclusions:** SRS+Sys was associated with longer survival compared to other treatment strategies. However, treatment selection is highly dependent on individual clinical factors such as performance status, comorbidities, and disease extent; therefore, these findings must be interpreted with caution Future prospective studies incorporating molecular and biomarker data are warranted to better guide care in this rare and high-risk group.

## Simple Summary

Brain metastases (BM) are an uncommon but grave stage of metastatic colorectal cancer (CRC) that significantly worsen patient outcomes. In this study, we analyzed data from the National Cancer Database to better understand survival patterns and treatment strategies among CRC patients diagnosed with BM between 2010 and 2020. We focused on patients whose cancer had spread only to the brain and found that those who received both systemic therapy (such as chemotherapy) and stereotactic radiosurgery (SRS), a precise form of radiation, had the longest survival. Patients who received no treatment had the poorest outcomes. Factors like age, type of primary tumor (colon vs. rectal), and access to surgery or radiation influenced outcomes, although overall health and insurance status were not strongly associated. These findings highlight the importance of individualized treatment plans and the need for more research to guide care for this high-risk but understudied group.

## 1. Introduction

More than 200,000 individuals are diagnosed with brain metastases (BM) annually in the United States (U.S.) [1]. In the U.S., BM represent the most frequent central nervous system (CNS) lesions. early 30% of patients with malignant tumors have BM [2,3]. In adults, common primary malignancies leading to BM are lung cancer, breast cancer, and melanoma, while colorectal cancer (CRC) accounts for only 2–5% of BM cases [4,5]. Magnetic resonance imaging (MRI) with and without contrast is used for BM diagnosis [6]. With the advancement of imaging modalities, there has been an increase in the reported incidence of BM in patients who have multiple metastases, including those with CRC [7]. However, the prognosis of CRC patients with BM remains poor, with a median survival of only 6–9 months [8]. Surgical intervention is often the most effective option to improve survival outcomes among patients [9,10]. While current literature sheds light on the clinical, radiological, and molecular characteristics of CRC-related BM [11], there are significant knowledge gaps in delayed detection and surgical interventions influencing treatment and survival outcomes.

Due to the rarity of BM in CRC patients, there is a lack of clinical data in the literature examining this population, as the focus is on more common metastatic sites such as the lungs and breast [12]. BM is associated with particularly poor prognoses, and the lack of populated-based data limits understanding of its epidemiology and management in CRC patients [13]. The reported incidence of CRC-related BM has gradually increased over the years, likely due to the advancements in imaging modalities and steadily increased uptake in systemic therapies (Sys) [14]. This study evaluates survival outcomes, treatment patterns, and prognostic factors among patients with CRC and BM, with specific attention to differences between those with isolated BM and those with concurrent extracranial metastases. Analyses focus on the influence of demographic, clinical, and treatment-related variables on overall survival and assess variation in treatment utilization across subgroups.

## 2. Materials and Methods

### 2.1. Study Design and Data Source

This retrospective study assesses CRC patients with isolated BM compared to those with extracranial metastases in the U.S. between 2010 and 2020. Data were obtained from the National Cancer Database (NCDB), which is a joint initiative of the American Cancer Society (ACS) and the American College of Surgeons’ Commission on Cancer (CoC). The database aggregates data from over 1500 accredited cancer programs, which has nearly 70% of newly diagnosed cancer cases nationwide [15]. The database comprises de-identified data on patient demographics, tumor characteristics, treatment patterns, and survival outcomes. ICD-O-3 and NCDB codes specific to this study are listed in Supplementary Table S1.

### 2.2. Study Population

This study included all patients diagnosed with CRC and isolated BM with extracranial metastasis (i.e., liver, lung, bone) serving as a comparative arm between 2010 and 2020. This study includes patients with synchronous metastases, those present and recorded at the time of initial CRC diagnosis. TNM staging data reflected the clinical stage at presentation and did not document changes resulting from treatment response or disease progression. Patients without BM or with only extracranial metastatic disease were excluded. The included sample did not have leptomeningeal or spinal metastases.Additional exclusions were applied for missing data on tumor grade or stage. All patients with BM, including those who did not receive cancer-directed therapies (radiotherapy, chemotherapy, or immunotherapy), were included in descriptive analyses to characterize the full population. Patients with missing key covariates or survival follow-up were also excluded from inferential analyses.

### 2.3. Variables and Covariates

The primary endpoint of this study was overall survival (OS), defined as the time from the diagnosis of BM to death or last known follow-up, as recorded in the NCDB. Survival status or mortality was determined based on whether a patient was alive or deceased at the time of last contact. Survival outcomes encompassed multiple metrics including overall survival, survival status, and time-to-event in months. Patients were categorized into two groups: those with isolated brain metastases (BM only, no evidence of extracranial metastatic disease) and those with concurrent extracranial metastases (i.e., additional metastases to organs including the liver, lung, or bone at diagnosis).

Treatment variables included receipt of stereotactic radiosurgery (SRS), whole-brain radiotherapy (WBRT), and systemic therapy (Sys), defined as the administration of chemotherapy and/or immunotherapy. These were grouped into six mutually exclusive categories to mirror real-world clinical combinations: SRS with Sys (SRS+Sys), WBRT with Sys (WBRT+Sys), SRS alone (without Sys), WBRT alone, Sys alone (without radiation), and no treatment (defined as receiving neither radiation nor Sys). Radiation modality was categorized as WBRT, SRS, or none. Sys was considered present if chemotherapy and/or immunotherapy were administered and absent otherwise. Surgical treatment was limited to the primary tumor site and categorized as no surgery versus any surgery of the primary site, the latter combining local excision and definitive resection due to dataset limitations on surgical detail at metastatic sites.

Covariates were grouped categorically for analysis. Age was stratified into two groups: 40–64 and 65–90 years. Sex was categorized as male or female; race as White or non-White; and ethnicity as Hispanic or non-Hispanic. Insurance status was grouped into private, public (including Medicare and Medicaid), or uninsured. Median household income was dichotomized based on the national distribution at <USD 74,063 and ≥USD 74,063. Educational attainment at the community level was stratified using the percentage of adults without a high school diploma, grouped as ≥9.1% vs. <9.1% (an NCDB-specific categorization). Facility type was categorized as academic (including NCI-designated centers and research hospitals) or non-academic. Geographic location was grouped into metropolitan vs. non-metropolitan based on the USDA rural-urban classification.

Clinical covariates included the Charlson–Deyo comorbidity index, grouped as 0, 1, and 2–3. Tumor size was categorized into <40 mm, 40–70 mm, and >70 mm in alignment with commonly used clinical thresholds [16,17]. Tumor grade was categorized from I to IV based on histologic differentiation. Primary disease site was defined as the colon or rectum. Year of diagnosis was grouped into two periods, 2010–2015 and 2016–2020, to account for evolving treatment practices over time.

### 2.4. Statistical Analysis

Descriptive statistics were calculated to summarize the demographic, clinical, and treatment-related characteristics of the study population. Continuous variables were reported as medians with interquartile ranges (IQRs), and categorical variables were described as frequencies and percentages. Unadjusted Kaplan-Meier survival curves were generated to visually depict OS, with comparisons made using the log-rank test;edian OS and survival rates at 3, 6, 12, 24, and 36 months were stratified by treatment modality and primary tumor site. To assess factors associated wth survival among patients with brain-only metastases, we employed multivariable logistic regression models using survival status at 6 months (alive vs. deceased) as the outcome, with supplementary analyses for time cutoffs at 12 months, 18 months and 24 months. Adjusted odds ratios (ORs) and 95% confidence intervals (CIs) were reported. Multivariable Cox proportional hazards regression models were used to evaluate time-to-event outcomes for OS. Models were constructed with treatment modalities entered as either individual variables or consolidated composite categories. Hazard ratios (HRs) with 95% CIs were reported for each covariate. Treatment modalities, education level, race, and insurance status were among the factors evaluated in the AFT framework. Univariate Cox regression analyses were initially conducted for all covariates. Variables with a *p*-value < 0.70 were considered for inclusion in multivariable models to account for potential confounders and preserved statistical power. Assumptions of proportional hazards were assessed using Schoenfeld residuals. Variables with significant time-varying effects were noted, and supplementary stratified or time-interaction models were considered where necessary. To further test the robustness of findings, AFT models were conducted using log-linear modeling. Time ratios (TRs) were computed to assess the relative effect of covariates on survival duration. All statistical tests were two-sided, with a significance threshold of *p* < 0.05. Analyses were performed using R statistical software (version 4.3.3; The R Foundation for Statistical Computing, Indianapolis, IN, USA). The study followed Strengthening the Reporting of Observational Studies in Epidemiology (STROBE) reporting guidelines.

## 3. Results

### 3.1. Cohort Identification and Study Population

**Figure 1:**
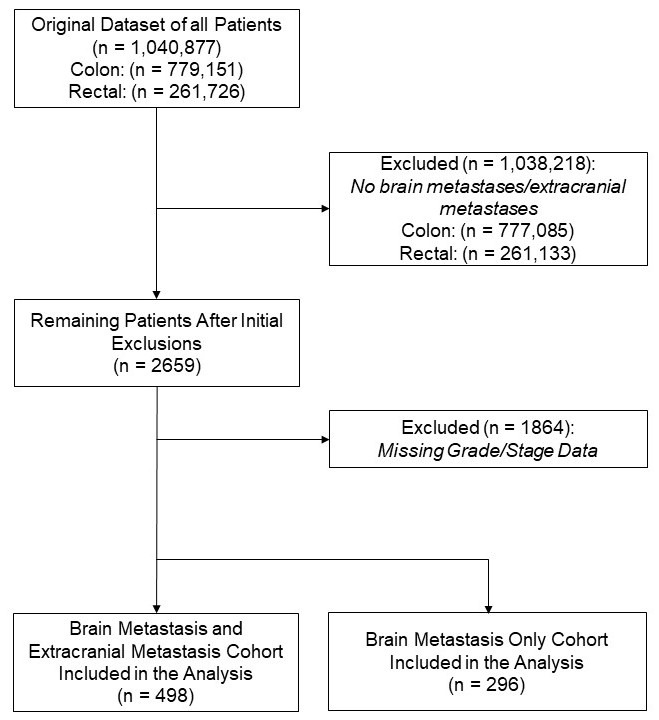
STROBE flow diagram of patient selection.

A total of 1,040,877 individuals diagnosed with colon (n = 779,151) or rectal (n = 261,726) cancer were identified. After excluding 1864 patients with missing grade or stage information, 795 patients were included with metastatic disease, of which 296 had isolated BM, comprising the primary analytic cohort for this study (Figure 1).

### 3.2. Baseline Characteristics

#### 3.2.1. By Metastatic Status (Brain-Only vs. Brain and Other Metastases)

Among 794 patients with metastatic CRC, 296 (37.3%) had brain-only metastases, and 498 (62.7%) had additional extracranial metastases. Median age was 67 years in the BM-only group and 64 years in the group with other metastases. A higher proportion of patients aged 65–90 years was observed in the BM-only group (58.4% vs. 48.0%). Female patients comprised 52.7% of the BM-only group and 48.2% of those with other metastases. Most patients in both groups were white (87.5% vs. 82.7%) and non-Hispanic (90.5% vs. 90.2%). Charlson–Deyo comorbidity scores, insurance status, median income, community education level, and facility type were similar across both groups. Tumor size greater than 70 mm was observed in 29.4% of the BM-only group and 19.5% of the other group. Grade III–IV tumors were present in 51.7% of BM-only cases compared to 33.3% in patients with additional metastases. Definitive surgery of primary site was performed in 73.3% of the BM-only group and 49.2% of the group with other metastases. SRS was administered to 22.6% and 16.9% of patients, respectively. Sys use was recorded in 52.4% of BM-only cases and 60.0% of those with other metastases. WBRT+Sys and Sys alone were more frequent in the group with extracranial disease (Table 1).

#### 3.2.2. By Primary Tumor Site (Colon vs. Rectum)

In total, 622 (78.3%) had colon cancer, and 172 (21.7%) had rectal cancer. The median age was 66 years in the colon group and 62 years in the rectum group. A greater proportion of rectal cancer patients were between 40 and 64 years old (57.6% vs. 45.5%). Gender, race, and ethnicity distributions were similar between groups. Rectal cancer patients had a higher proportion with no comorbid conditions (77.3% vs. 71.9%) and were more likely to have private insurance (41.3% vs. 32.2%) and reside in higher-income areas (59.9% with median income ≥ USD 74,063 vs. 32.0%). Academic center care was more frequent in rectal cases (37.8% vs. 28.1%). Grade II tumors predominated in both groups but were more frequent in rectal cancer (64.5% vs. 50.5%), while grade III tumors were more common in colon cancer (37.8% vs. 26.7%). Regarding metastasis patterns, brain-only disease was observed in 38.6% of colon and 32.6% of rectal cancer patients. Rectal cancer patients were more likely to receive Sys (70.9% vs. 53.4%) and less likely to undergo definitive surgery of the primary site (25.6% vs. 67.2%). Use of WBRT+Sys and Sys only was more frequent among rectal cases, while SRS and definitive local treatments were more common in colon cancer (Table 2).

### 3.3. Brain-Metastasis-Only Patients

Among the BM-only patients, 240 (81.1%) had colon cancer, and 56 (18.9%) had rectal cancer. Rectal cancer patients were more likely to be younger, privately insured, and reside in higher-income and more educated communities. Definitive surgery of the primary site was more common in colon cancer, while Sys alone was more frequently used in rectal cancer (Table 3).

When stratified by survival status, survivors tended to be younger and more likely to have received SRS, definitive surgery of the primary site, or multimodal treatment. Patients who died were more likely to have received WBRT alone or no treatment (Table 4).

### 3.4. Logistic Regression Analysis of Survival

To identify factors associated with survival among patients with brain-only metastases, we performed multivariable logistic regression modeling. The outcome was vital status at 6-months (alive vs. deceased) in the BM-only cohort (N = 296).

Rectal cancer was independently associated with improved 6-month survival compared to colon cancer (OR 0.20, 95% CI 0.07–0.53, *p* = 0.002). Higher comorbidity burden (Charlson-Deyo 2–3: OR 4.24, *p* = 0.008) and Grade IV tumors (OR 12.43, *p* = 0.017) were linked to significantly worse odds of survival. Age, sex, race, income, and insurance were not significant in adjusted models.

**Figure 2:**
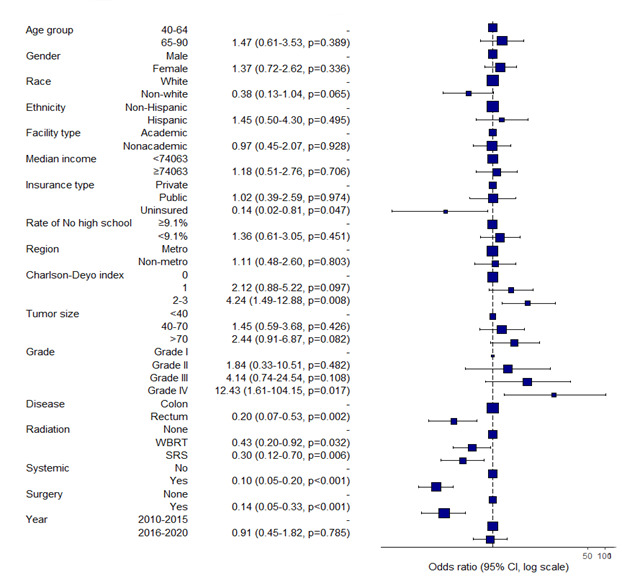
Adjusted odds ratios for survival in brain-metastasis-only patients at 6 months. Results are presented as Odds Ratio (95% Confidence Intervals, *p* value). Abbreviations: SRS: stereotactic radiosurgery; WBRT: whole-brain radiotherapy; Sys/Systemic: systemic therapy; CDI: Charlson–Deyo Index; Metro: metropolitan; Non-metro: non-metropolitan; HS: high school; Year: year of diagnosis (2010−2015 vs. 2016−2020). Surgrey referes to the primary site.

Full results are presented in Supplementary Table S2 and depicted in Figure 2. Additional logistic regression analyses for cutoffs at 12 months, 18 months and 24 months are presented in the Supplementary Materials (Supplementary Tables S3–S5; Supplementary Figures S1–S3).

### 3.5. Cox Proportional Hazards Modeling (Overall Survival)

To further evaluate factors associated with overall survival in patients with brain-only metastases (n = 296), we conducted multivariable Cox proportional hazards analyses. The primary outcome was time from diagnosis of brain metastases to death or last follow-up. The full model using separate treatment variables is presented in Table 5.

In the multivariable Cox model, several treatment modalities were independently associated with a lower risk of death. Demographic and socioeconomic factors such as age ≥ 65 years (HR = 1.41, 95% CI: 0.99–2.00, *p* = 0.054) and residence in areas with lower educational attainment (HR = 1.44, 95% CI: 1.03–2.02, *p* = 0.034) were associated with higher mortality.

Additional results including models using consolidated treatment categories and reduced covariate specifications are detailed in the Supplementary Materials (Supplementary Tables S6–S10).

### 3.6. Kaplan–Meier Estimates and Survival Rates

Kaplan–Meier survival analyses were conducted to evaluate overall survival in the brain-only metastases cohort (N = 296), stratified by treatment strategy and by primary cancer site. Median survival estimates and survival rates at 3 months, 6 months, 1 year, 2 years, and 3 years are summarized below.

Survival outcomes varied significantly by treatment strategy. Among brain-only cases, patients with rectal cancer had longer median survival than those with colon cancer (10.35 months [95% CI: 8.87–16.03] vs. 6.08 months [95% CI: 5.19–9.30]), though long-term survival rates were similar. When comparing across metastatic burden, patients with isolated brain metastases (N = 296) had significantly better survival than those with additional extracranial disease (N = 498), with median overall survival of 7.82 months (95% CI: 5.82–9.66) versus 5.29 months (95% CI: 4.53–6.41), respectively.

**Figure 3:**
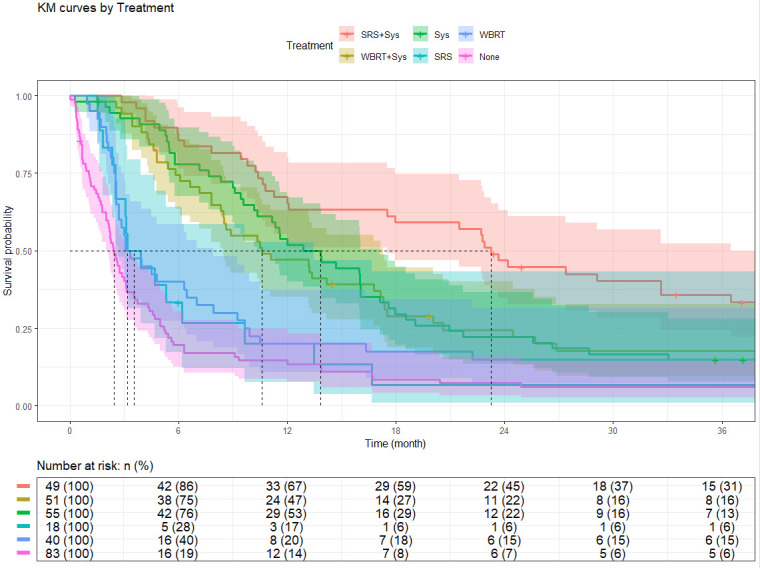
Kaplan–Meier curves by treatment. Abbreviations: KM: Kaplan–Meier; SRS: stereotactic radiosurgery; WBRT: whole-brain radiotherapy; Sys: systemic therapy; SRS+Sys: stereotactic radiosurgery combined with systemic therapy; WBRT+Sys: whole-brain radiotherapy combined with systemic therapy; None: no recorded cancer-directed therapy.

**Figure 4:**
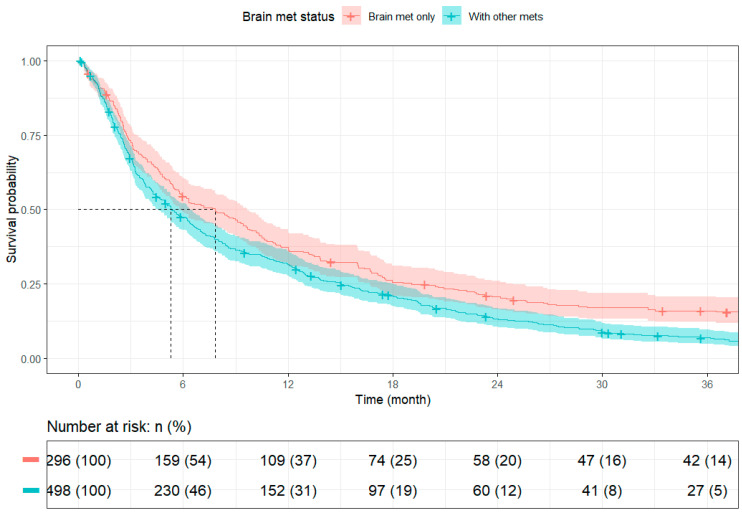
Kaplan–Meier curves by Metastatic Statu. Abbreviations: Brain met: brain metastasis; Mets: metastases; n: number at risk; %: percentage of initial cohort remaining at risk.

Detailed survival estimates are presented in Table 6 and Table 7, with corresponding Kaplan–Meier curves shown in Figure 3 and Figure 4.

### 3.7. Supplementary Analyses

Proportional hazards assumptions were evaluated via Schoenfeld residuals for each covariate. While most demographic and clinical variables conformed well to model assumptions, several treatment-related factors particularly radiation modality, Sys, and composite treatment categories displayed evidence of time-varying effects. These patterns suggest potential non-proportional hazards, warranting cautious interpretation of those variables in the Cox models. Diagnostic plots for all assessed covariates are presented in Supplementary Figures S4–S15.

To assess the robustness of our survival findings and explore potential deviations from modeling assumptions, we conducted supplementary analyses using accelerated failure time (AFT) models and proportional hazards diagnostics. Two AFT models were estimated: one incorporating treatment modalities as separate variables (surgery, Sys, and radiation; Supplementary Table S11) and another using a composite treatment variable (Supplementary Table S12). Across both models, receipt of definitive surgery of the primary site, SRS, and Sys was consistently associated with longer survival durations. Socioeconomic indicators such as education level and insurance type, along with race, also emerged as significant variables.

## 4. Discussion

This study provides a comprehensive national analysis of CRC patients with BM, identifying key clinicalfactors and treatment strategies associated with survival. While prior research has focused largely on more common metastatic sites such as the lungs and breast, data on CRC-related BM remain limited.

In our cohort of 794 metastatic CRC patients, 37.2% presented with isolated BM. These patients demonstrated longer median survival and were more likely to undergo definitive surgery of the primary site and receive SRS, suggesting that isolated BM may represent a clinically distinct subgroup, whereas patients with extracranial metastases were more likely to receive Sys alone. These findings align with the existing literature reporting less favorable survival among patients with extracranial or multiple metastatic sites [18,19,20,21]. While older age (≥65 years) was associated with worse survival in unadjusted analyses, it did not reach statistical significance in the multivariable model, contrasting with prior studies [16,17,18,19]. Jeri-Yabar et al. (2024) reported that individuals aged ≥50 years had a 1.58-fold higher risk of all-cause mortality compared to younger patients (HR = 1.58, 95% CI: 1.01–2.46, *p* = 0.045) using SEER data [22]. Our study found that patients with extracranial metastases had significantly shorter median survival compared to those with isolated BM (5.29 vs. 7.82 months), reinforcing prior evidence that greater metastatic burden is associated with poorer outcomes. Bonadio et al. (2021) similarly reported reduced survival in patients with multiple BM (HR: 1.62, *p* = 0.001) [23]. These findings highlight the prognostic importance of both disease extent and patient age. In addition, We also observed that patients with rectal cancer had longer median survival than those with colon cancer (10.35 vs. 6.08 months), suggesting a more favorable short-term prognosis in rectal primaries among BM patients and with prior reports suggesting biologic and therapeutic differences by primary tumor site [24,25,26].

In our study, gender was not significantly associated with OS among CRC patients with BM-only metastases. In adjusted Cox proportional hazards models, female patients had a hazard ratio of 1.09 (95% CI: 0.84–1.42, *p* = 0.526) compared to males, indicating no significant difference in survival outcomes by sex. These findings do not align with prior research by Bonadio et al. (2021), which identified male gender as a negative prognostic factor in CRC patients with BM, reporting a 46% higher mortality risk among men (HR: 1.46; 95% CI: 1.08–1.97; *p* = 0.012) [23]. The underlying mechanisms for sex-based differences in prognosis remain poorly understood.

High comorbidity burden led to higher 6-month mortality (Charlson-Deyo index of 2–3; OR = 4.24, 95% CI: 1.49–12.88, *p* = 0.008), however, moderate burden (Charlson-Deyo index of 1; OR = 2.12, 95% CI: 0.88–5.22, *p* = 0.097) did not impact mortality (*p* = 0.097). In adjusted Cox models, neither moderate (Charlson–Deyo index of 1; HR = 1.34, 95% CI: 0.93–1.93, *p* = 0.113) nor high comorbidity burden (Charlson–Deyo index of 2–3; HR = 1.24, 95% CI: 0.81–1.89, *p* = 0.326) demonstrated an independent association with increased mortality risk. Current literature reports such as those by Cooper et al. (2021) found a nearly threefold increase in mortality among all cancer patients with ≥5 comorbidities (HR: 3.39, *p* < 0.001) [27]. The variation may reflect differences in patient population, comorbidity distribution, or the unique clinical trajectory of brain-only metastases in CRC. Nonetheless, our study documentedthat the presence of extracranial metastases was associated with significantly shorter survival compared to isolated BM (median OS: 5.29 vs. 7.82 months), consistent with prior findings by Gao et al. (2023) and Koo et al. (2020) that highlight the adverse impact of greater metastatic burden [8,28]. While comorbidity burden did not emerge as a significant prognostic factor in this subset, it may still influence therapeutic decisions and outcomes in broader or more heterogeneous CRC populations.

Health access and insurance coverage were evaluated as factors associated with survival in the current study. While unadjusted analyses showed differences in survival by insurance type, with higher crude survival among privately insured patients, these differences were not significant in adjusted models. Specifically, public insurance (HR = 1.13, 95% CI: 0.77–1.65, *p* = 0.536) and uninsured status (HR = 0.73, 95% CI: 0.41–1.30, *p* = 0.288) were not independently associated with OS compared to private insurance. These findings suggest that insurance status alone may not fully account for disparities in survival outcomes within this cohort. However, prior research such as that by Wray and colleagues reported that private insurance may offer broader options but also presents challenges related to cost and continuity of care [29]. In contrast, non-private insurance plans often provide more cost-efficient care but may limit access to novel therapies due to reimbursement limitations. Wallace et al. further described how Medicare’s purchasing power contributes to lower spending through reduced provider prices, though this can restrict coverage flexibility, particularly for patients with advanced cancer [30]. These structural differences highlight the ongoing need to address imbalances across insurance systems and ensure equitable access to clinical care for patients with CRC and BM [31].

Treatment approach was significantly associated with OS among patients with brain-only metastases. In this cohort, the longest median survival was observed in patients treated with SRS+Sys, who had a median OS of 23.26 months and a 3-year survival rate of 35.8%. Patients who did not receive any recorded cancer-directed therapy had a median survival of 2.43 months. Intermediate outcomes were noted for those receiving Sys alone (13.83 months) and WBRT+Sys (10.61 months), whereas single-modality radiotherapy was associated with shorter survival durations. While these findings are consistent with previous studies reporting improved outcomes with select combination treatments in CRC patients with BM [23,32,33,34,35,36], they should be interpreted within the context of individual patient characteristics. Treatment selection is influenced by multiple clinical considerations, including functional status, comorbidity profile, extent of disease, and patient preferences. As such, treatment selection data, such as performance status, neurologic symptoms, or intent of care (curative vs. palliative), are not documented in the NCDB, limiting the ability to determine the rationale behind therapy choice. Thus, observed associations between treatment modality and survival should be interpreted as correlational rather than causal and likely reflect a combination of clinical appropriateness, access to care, and patient selection factors.

### 4.1. Limitations

This study benefits from the national scale of the NCDB but is subject to limitations inherent to retrospective registry analyses. The NCDB lacks granular molecular data and detailed treatment parameters, restricting assessment of personalized therapies, with systemic therapy data limited to binary receipt indicators without details on agents, intensity, or completion. Treatment intent (curative vs. palliative) was not recorded. Key clinical variables such as performance status, number or volume of brain metastases, extent of surgical resection, and radiation dose were not available, and only patients with synchronous brain metastases were included.

### 4.2. Future Directions

While subject to certain limitations, this study seeks to be a valuable tool for examining real-world, population-level trends in rare cancer subsets such as CRC-BM. This study provides necessary insights into survival patterns, treatment disparities, and practice variation over a decade-long period. Given the rarity of CRC-BM, our findings highlight the need for prospective, biomarker-driven studies that incorporate performance status, BMdisease burden, and detailed treatment variables to better guide therapy selection. Future research should also explore access-related barriers, especially for high-resource treatments like SRS, and prioritize the development of tailored clinical trials for this high-risk population.

## 5. Conclusions

In this analysis of the U.S. NCDB, patients with isolated synchronous BM had better outcomes and were more frequently treated with surgery to the primary site and localized brain-directed radiotherapy, suggesting a clinically distinct subgroup with more favorable disease biology or access to specialized care. The combination of SRS and systemic therapy was associated with the longest survival, while definitive surgery of the primary site, SRS, and systemic therapy each independently conferred survival benefit. Patients with isolated synchronous BM had better outcomes and were more frequently treated with surgery to the primary site These findings, though limited by retrospective design and lack of data on performance status or intracranial burden, support aggressive multimodal therapy and highlight the need for prospective studies to guide individualized care.

## Figures and Tables

**Table 1:** Baseline demographic and clinical characteristics by the presence of extracranial metastases among patients with brain metastases from colorectal cancer (N = 794).

Group	Level	Brain Metastases Only (n = 296)	With Other Metastasis (n = 498)	All (N = 794)
**Age**	Median (range)	67 (40 to 90)	64 (40 to 90)	65 (40 to 90)
**Age group**	40–64 years	123 (41.6)	259 (52.0)	382 (48.1)
	65–90 years	173 (58.4)	239 (48.0)	412 (51.9)
**Gender**	Male	140 (47.3)	258 (51.8)	398 (50.1)
	Female	156 (52.7)	240 (48.2)	396 (49.9)
**Race**	White	259 (87.5)	412 (82.7)	671 (84.5)
	Non-white	37 (12.5)	86 (17.3)	123 (15.5)
**Ethnicity**	Non-Hispanic	268 (90.5)	449 (90.2)	717 (90.3)
	Hispanic	28 (9.5)	49 (9.8)	77 (9.7)
**Charlson–Deyo index**	0	220 (74.3)	360 (72.3)	580 (73.0)
	1	43 (14.5)	90 (18.1)	133 (16.8)
	2–3	33 (11.1)	48 (9.6)	81 (10.2)
**Insurance type**	Private	90 (30.4)	181 (36.3)	271 (34.1)
	Public	186 (62.8)	282 (56.6)	468 (58.9)
	Uninsured	20 (6.8)	35 (7.0)	55 (6.9)
**Median income**	<USD 74,063	182 (61.5)	310 (62.2)	492 (62.0)
	≥USD 74,063	114 (38.5)	188 (37.8)	302 (38.0)
**Rate of no high school completion**	≥9.1%	158 (53.4)	250 (50.2)	408 (51.4)
	<9.1%	138 (46.6)	248 (49.8)	386 (48.6)
**Region**	Metropolitan	232 (78.4)	416 (83.5)	648 (81.6)
	Non-metropolitan	64 (21.6)	82 (16.5)	146 (18.4)
**Facility type**	Academic	90 (30.4)	150 (30.1)	240 (30.2)
	Non-academic	206 (69.6)	348 (69.9)	554 (69.8)
**Tumor size**	<40 mm	58 (19.6)	128 (25.7)	186 (23.4)
	40–70 mm	151 (51.0)	273 (54.8)	424 (53.4)
	>70 mm	87 (29.4)	97 (19.5)	184 (23.2)
**Grade**	Grade I	14 (4.7)	36 (7.2)	50 (6.3)
	Grade II	129 (43.6)	296 (59.4)	425 (53.5)
	Grade III	131 (44.3)	150 (30.1)	281 (35.4)
	Grade IV	22 (7.4)	16 (3.2)	38 (4.8)
**Histology type**	Adenocarcinomas	278 (93.9)	453 (91.0)	731 (92.1)
	Non-adenocarcinomas	18 (6.1)	45 (9.0)	63 (7.9)
**Year**	2010–2015	186 (62.8)	297 (59.6)	483 (60.8)
	2016–2020	110 (37.2)	201 (40.4)	311 (39.2)
**Disease**	Colon	240 (81.1)	382 (76.7)	622 (78.3)
	Rectum	56 (18.9)	116 (23.3)	172 (21.7)
**Radiation**	None	138 (46.6)	260 (52.2)	398 (50.1)
	WBRT	91 (30.7)	154 (30.9)	245 (30.9)
	SRS	67 (22.6)	84 (16.9)	151 (19.0)
**Systemic**	No	141 (47.6)	199 (40.0)	340 (42.8)
	Yes	155 (52.4)	299 (60.0)	454 (57.2)
**Surgery of Primary Site**	None	77 (26.0)	247 (49.6)	324 (40.8)
	Local excision	2 (0.7)	6 (1.2)	8 (1.0)
	Definitive	217 (73.3)	245 (49.2)	462 (58.2)
**Treatment**	SRS+Sys	49 (16.6)	62 (12.4)	111 (14.0)
	WBRT+Sys	51 (17.2)	104 (20.9)	155 (19.5)
	Sys	55 (18.6)	133 (26.7)	188 (23.7)
	SRS	18 (6.1)	22 (4.4)	40 (5.0)
	WBRT	40 (13.5)	50 (10.0)	90 (11.3)
	None	83 (28.0)	127 (25.5)	210 (26.4)

Abbreviations: WBRT: whole-brain radiotherapy; SRS: stereotactic radiosurgery; Sys: systemic therapy; SRS+Sys: stereotactic radiosurgery combined with systemic therapy; WBRT+Sys: whole-brain radiotherapy combined with systemic therapy; CDI: Charlson–Deyo Index; <9.1%: less than 9.1% of adults in the patient’s residential area did not complete high school; ≥9.1%: 9.1% or more of adults in the area did not complete high school.

**Table 2:** Demographic characteristics by cancer type (N = 794).

Group	Level	Colon (N = 622)	Rectum (N = 172)	All (N = 794)
**Age**	Median (range)	66 (40 to 90)	62 (40 to 90)	65 (40 to 90)
**Age group**	40–64 years	283 (45.5)	99 (57.6)	382 (48.1)
	65–90 years	339 (54.5)	73 (42.4)	412 (51.9)
**Gender**	Male	306 (49.2)	92 (53.5)	398 (50.1)
	Female	316 (50.8)	80 (46.5)	396 (49.9)
**Race**	White	525 (84.4)	146 (84.9)	671 (84.5)
	Non-white	97 (15.6)	26 (15.1)	123 (15.5)
**Ethnicity**	Non-Hispanic	558 (89.7)	159 (92.4)	717 (90.3)
	Hispanic	64 (10.3)	13 (7.6)	77 (9.7)
**Charlson–Deyo index**	0	447 (71.9)	133 (77.3)	580 (73.0)
	1	104 (16.7)	29 (16.9)	133 (16.8)
	2–3	71 (11.4)	10 (5.8)	81 (10.2)
**Insurance type**	Private	200 (32.2)	71 (41.3)	271 (34.1)
	Public	382 (61.4)	86 (50.0)	468 (58.9)
	Uninsured	40 (6.4)	15 (8.7)	55 (6.9)
**Median income**	<USD 74,063	423 (68.0)	69 (40.1)	492 (62.0)
	≥USD 74,063	199 (32.0)	103 (59.9)	302 (38.0)
**Rate of no high school completion**	≥9.1%	339 (54.5)	69 (40.1)	408 (51.4)
	<9.1%	283 (45.5)	103 (59.9)	386 (48.6)
**Region**	Metropolitan	501 (80.5)	147 (85.5)	648 (81.6)
	Non-metropolitan	121 (19.5)	25 (14.5)	146 (18.4)
**Facility type**	Academic	175 (28.1)	65 (37.8)	240 (30.2)
	Non-academic	447 (71.9)	107 (62.2)	554 (69.8)
**Tumor size**	<40 mm	140 (22.5)	46 (26.7)	186 (23.4)
	40–70 mm	337 (54.2)	87 (50.6)	424 (53.4)
	>70 mm	145 (23.3)	39 (22.7)	184 (23.2)
**Grade**	Grade I	41 (6.6)	9 (5.2)	50 (6.3)
	Grade II	314 (50.5)	111 (64.5)	425 (53.5)
	Grade III	235 (37.8)	46 (26.7)	281 (35.4)
	Grade IV	32 (5.1)	6 (3.5)	38 (4.8)
**Histology type**	Adenocarcinomas	578 (92.9)	153 (89.0)	731 (92.1)
	Non-adenocarcinomas	44 (7.1)	19 (11.0)	63 (7.9)
**Year**	2010–2015	384 (61.7)	99 (57.6)	483 (60.8)
	2016–2020	238 (38.3)	73 (42.4)	311 (39.2)
**Metastatic status**	Brain metastasis only	240 (38.6)	56 (32.6)	296 (37.3)
	With other metastases	382 (61.4)	116 (67.4)	498 (62.7)
**Radiation**	None	306 (49.2)	92 (53.5)	398 (50.1)
	WBRT	196 (31.5)	49 (28.5)	245 (30.9)
	SRS	120 (19.3)	31 (18.0)	151 (19.0)
**Systemic**	No	290 (46.6)	50 (29.1)	340 (42.8)
	Yes	332 (53.4)	122 (70.9)	454 (57.2)
**Surgery of Primary Site**	None	202 (32.5)	122 (70.9)	324 (40.8)
	Local excision	2 (0.3)	6 (3.5)	8 (1.0)
	Definitive	418 (67.2)	44 (25.6)	462 (58.2)
**Treatment**	SRS+Sys	86 (13.8)	25 (14.5)	111 (14.0)
	WBRT+Sys	117 (18.8)	38 (22.1)	155 (19.5)
	Sys	129 (20.7)	59 (34.3)	188 (23.7)
	SRS	34 (5.5)	6 (3.5)	40 (5.0)
	WBRT	79 (12.7)	11 (6.4)	90 (11.3)
	None	177 (28.5)	33 (19.2)	210 (26.4)

Abbreviations: WBRT: whole-brain radiotherapy; SRS: stereotactic radiosurgery; Sys: systemic therapy; SRS+Sys: stereotactic radiosurgery combined with systemic therapy; WBRT+Sys: whole-brain radiotherapy combined with systemic therapy; CDI: Charlson–Deyo Index; <9.1%: less than 9.1% of adults in the patient’s residential area did not complete high school; ≥9.1%: 9.1% or more of adults in the area did not complete high school; Academic: academic medical center; Non-academic: community or non-academic facility.

**Table 3:** Demographic characteristics in brain-metastasis-only patients by primary cancer type (N = 296).

Variable	Group	Colon (N = 240)	Rectum (N = 56)	All (N = 296)
**Age**	Median (range)	68 (40 to 90)	62 (41 to 90)	67 (40 to 90)
**Age group**	40–64 years	92 (38.3)	31 (55.4)	123 (41.6)
	65–90 years	148 (61.7)	25 (44.6)	173 (58.4)
**Gender**	Male	108 (45.0)	32 (57.1)	140 (47.3)
	Female	132 (55.0)	24 (42.9)	156 (52.7)
**Race**	White	209 (87.1)	50 (89.3)	259 (87.5)
	Non-white	31 (12.9)	6 (10.7)	37 (12.5)
**Ethnicity**	Non-Hispanic	217 (90.4)	51 (91.1)	268 (90.5)
	Hispanic	23 (9.6)	5 (8.9)	28 (9.5)
**Charlson–Deyo index**	0	175 (72.9)	45 (80.4)	220 (74.3)
	1	36 (15.0)	7 (12.5)	43 (14.5)
	2–3	29 (12.1)	4 (7.1)	33 (11.1)
**Insurance type**	Private	65 (27.1)	25 (44.6)	90 (30.4)
	Public	161 (67.1)	25 (44.6)	186 (62.8)
	Uninsured	14 (5.8)	6 (10.7)	20 (6.8)
**Median income**	<USD 74,063	164 (68.3)	18 (32.1)	182 (61.5)
	≥USD 74,063	76 (31.7)	38 (67.9)	114 (38.5)
**Rate of no high school completion**	≥9.1%	140 (58.3)	18 (32.1)	158 (53.4)
	<9.1%	100 (41.7)	38 (67.9)	138 (46.6)
**Region**	Metropolitan	184 (76.7)	48 (85.7)	232 (78.4)
	Non-metropolitan	56 (23.3)	8 (14.3)	64 (21.6)
**Facility type**	Academic	71 (29.6)	19 (33.9)	90 (30.4)
	Non-academic	169 (70.4)	37 (66.1)	206 (69.6)
**Tumor size**	<40 mm	45 (18.8)	13 (23.2)	58 (19.6)
	40–70 mm	123 (51.2)	28 (50.0)	151 (51.0)
	>70 mm	72 (30.0)	15 (26.8)	87 (29.4)
**Grade**	Grade I	12 (5.0)	2 (3.6)	14 (4.7)
	Grade II	98 (40.8)	31 (55.4)	129 (43.6)
	Grade III	110 (45.8)	21 (37.5)	131 (44.3)
	Grade IV	20 (8.3)	2 (3.6)	22 (7.4)
**Histology type**	Adenocarcinomas	225 (93.8)	53 (94.6)	278 (93.9)
	Non-adenocarcinomas	15 (6.2)	3 (5.4)	18 (6.1)
**Year**	2010–2015	148 (61.7)	38 (67.9)	186 (62.8)
	2016–2020	92 (38.3)	18 (32.1)	110 (37.2)
**Radiation therapy**	None	102 (42.5)	36 (64.3)	138 (46.6)
	WBRT	79 (32.9)	12 (21.4)	91 (30.7)
	SRS	59 (24.6)	8 (14.3)	67 (22.6)
**Systemic therapy**	No	124 (51.7)	17 (30.4)	141 (47.6)
	Yes	116 (48.3)	39 (69.6)	155 (52.4)
**Surgery of Primary Site**	None	51 (21.2)	26 (46.4)	77 (26.0)
	Local excision	0 (0.0)	2 (3.6)	2 (0.7)
	Definitive	189 (78.8)	28 (50.0)	217 (73.3)
**Treatment**	SRS+Sys	42 (17.5)	7 (12.5)	49 (16.6)
	WBRT+Sys	43 (17.9)	8 (14.3)	51 (17.2)
	Sys	31 (12.9)	24 (42.9)	55 (18.6)
	SRS	17 (7.1)	1 (1.8)	18 (6.1)
	WBRT	36 (15.0)	4 (7.1)	40 (13.5)
	None	71 (29.6)	12 (21.4)	83 (28.0)

Abbreviations: WBRT: whole-brain radiotherapy; SRS: stereotactic radiosurgery; Sys: systemic therapy; SRS+Sys: stereotactic radiosurgery combined with systemic therapy; WBRT+Sys: whole-brain radiotherapy combined with systemic therapy; CDI: Charlson–Deyo Index; <9.1%: less than 9.1% of adults in the patient’s residential area did not complete high school; ≥9.1%: 9.1% or more of adults in the area did not complete high school; Academic: academic medical center; Non-academic: community or non-academic facility; 2010–2015/2016–2020: year of diagnosis grouped for temporal comparison.

**Table 4:** Descriptive findings in brain-metastasis-only patients by survival status (N = 296).

Variable	Group	Alive (N = 34)	Dead (N = 262)	All (N = 296)
**Age**	Median (range)	63 (40 to 89)	68 (40 to 90)	67 (40 to 90)
**Age group**	40–64 years	21 (61.8)	102 (38.9)	123 (41.6)
	65–90 years	13 (38.2)	160 (61.1)	173 (58.4)
**Gender**	Male	18 (52.9)	122 (46.6)	140 (47.3)
	Female	16 (47.1)	140 (53.4)	156 (52.7)
**Race**	White	27 (79.4)	232 (88.5)	259 (87.5)
	Non-white	7 (20.6)	30 (11.5)	37 (12.5)
**Ethnicity**	Non-Hispanic	31 (91.2)	237 (90.5)	268 (90.5)
	Hispanic	3 (8.8)	25 (9.5)	28 (9.5)
**Charlson–Deyo index**	0	28 (82.4)	192 (73.3)	220 (74.3)
	1	3 (8.8)	40 (15.3)	43 (14.5)
	2–3	3 (8.8)	30 (11.5)	33 (11.1)
**Insurance type**	Private	14 (41.2)	76 (29.0)	90 (30.4)
	Public	15 (44.1)	171 (65.3)	186 (62.8)
	Uninsured	5 (14.7)	15 (5.7)	20 (6.8)
**Median income**	<USD 74,063	23 (67.6)	159 (60.7)	182 (61.5)
	≥USD 74,063	11 (32.4)	103 (39.3)	114 (38.5)
**Rate of no high school completion**	≥9.1%	23 (67.6)	135 (51.5)	158 (53.4)
	<9.1%	11 (32.4)	127 (48.5)	138 (46.6)
**Region**	Metropolitan	26 (76.5)	206 (78.6)	232 (78.4)
	Non-metropolitan	8 (23.5)	56 (21.4)	64 (21.6)
**Facility type**	Academic	9 (26.5)	81 (30.9)	90 (30.4)
	Non-academic	25 (73.5)	181 (69.1)	206 (69.6)
**Tumor size**	<40 mm	8 (23.5)	50 (19.1)	58 (19.6)
	40–70 mm	16 (47.1)	135 (51.5)	151 (51.0)
	>70 mm	10 (29.4)	77 (29.4)	87 (29.4)
**Grade**	Grade I	1 (2.9)	13 (5.0)	14 (4.7)
	Grade II	20 (58.8)	109 (41.6)	129 (43.6)
	Grade III	12 (35.3)	119 (45.4)	131 (44.3)
	Grade IV	1 (2.9)	21 (8.0)	22 (7.4)
**Histology type**	Adenocarcinomas	34 (100.0)	244 (93.1)	278 (93.9)
	Non-adenocarcinomas	0 (0.0)	18 (6.9)	18 (6.1)
**Year**	2010–2015	16 (47.1)	170 (64.9)	186 (62.8)
	2016–2020	18 (52.9)	92 (35.1)	110 (37.2)
**Disease**	Colon	30 (88.2)	210 (80.2)	240 (81.1)
	Rectum	4 (11.8)	52 (19.8)	56 (18.9)
**Radiation therapy**	None	10 (29.4)	128 (48.9)	138 (46.6)
	WBRT	9 (26.5)	82 (31.3)	91 (30.7)
	SRS	15 (44.1)	52 (19.8)	67 (22.6)
**Systemic therapy**	No	9 (26.5)	132 (50.4)	141 (47.6)
	Yes	25 (73.5)	130 (49.6)	155 (52.4)
**Surgery of Primary Site**	None	3 (8.8)	74 (28.2)	77 (26.0)
	Local excision	0 (0.0)	2 (0.8)	2 (0.7)
	Definitive	31 (91.2)	186 (71.0)	217 (73.3)
**Treatment**	SRS+Sys	14 (41.2)	35 (13.4)	49 (16.6)
	WBRT+Sys	6 (17.6)	45 (17.2)	51 (17.2)
	Sys	5 (14.7)	50 (19.1)	55 (18.6)
	SRS	1 (2.9)	17 (6.5)	18 (6.1)
	WBRT	3 (8.8)	37 (14.1)	40 (13.5)
	None	5 (14.7)	78 (29.8)	83 (28.0)

Abbreviations: WBRT: whole-brain radiotherapy; SRS: stereotactic radiosurgery; Sys: systemic therapy; SRS+Sys: stereotactic radiosurgery combined with systemic therapy; WBRT+Sys: whole-brain radiotherapy combined with systemic therapy; CDI: Charlson–Deyo Index; <9.1%: less than 9.1% of adults in the patient’s residential area did not complete high school; ≥9.1%: 9.1% or more of adults in the area did not complete high school; Academic: academic medical center; Non-academic: community or non-academic facility; 2010–2015/2016–2020: year of diagnosis grouped for temporal comparison; Alive/Dead: vital status at last follow-up.

**Table 5:** Cox proportional hazards analysis (full model with separate treatment variables).

Variable	Group	N (%)	HR (Univariable)	HR (Multivariable)
Age group	40–64 years	123 (41.6)	-	-
	65–90 years	173 (58.4)	1.98 (1.54–2.55, *p* < 0.001)	1.41 (0.99–2.00, *p* = 0.054)
Gender	Male	140 (47.3)	-	-
	Female	156 (52.7)	1.09 (0.86–1.39, *p* = 0.484)	1.09 (0.84–1.42, *p* = 0.526)
Race	White	259 (87.5)	-	-
	Non-white	37 (12.5)	0.81 (0.55–1.18, *p* = 0.272)	0.63 (0.41–0.97, *p* = 0.035)
Ethnicity	Non-Hispanic	268 (90.5)	-	-
	Hispanic	28 (9.5)	0.99 (0.65–1.49, *p* = 0.945)	0.88 (0.57–1.38, *p* = 0.587)
Facility type	Academic	90 (30.4)	-	-
	Non-academic	206 (69.6)	1.09 (0.84–1.41, *p* = 0.530)	1.17 (0.86–1.59, *p* = 0.309)
Median income	<USD 74,063	182 (61.5)	-	-
	≥USD 74,063	114 (38.5)	1.10 (0.86–1.41, *p* = 0.453)	0.78 (0.54–1.14, *p* = 0.205)
Insurance type	Private	90 (30.4)	-	-
	Public	186 (62.8)	1.72 (1.30–2.26, *p* < 0.001)	1.13 (0.77–1.65, *p* = 0.536)
	Uninsured	20 (6.8)	0.71 (0.41–1.24, *p* = 0.234)	0.73 (0.41–1.30, *p* = 0.288)
Rate of No high school	≥9.1%	158 (53.4)	-	-
	<9.1%	138 (46.6)	1.26 (0.99–1.61, *p* = 0.065)	1.44 (1.03–2.02, *p* = 0.034)
Region	Metropolitan	232 (78.4)	-	-
	Non-metropolitan	64 (21.6)	0.95 (0.71–1.28, *p* = 0.748)	0.94 (0.66–1.33, *p* = 0.725)
Charlson–Deyo index	0	220 (74.3)	-	-
	1	43 (14.5)	1.32 (0.94–1.86, *p* = 0.111)	1.34 (0.93–1.93, *p* = 0.113)
	2–3	33 (11.1)	1.54 (1.04–2.26, *p* = 0.029)	1.24 (0.81–1.89, *p* = 0.326)
Tumor size	<40 mm	58 (19.6)	-	-
	40–70 mm	151 (51.0)	1.10 (0.79–1.52, *p* = 0.585)	1.00 (0.70–1.42, *p* = 0.999)
	>70 mm	87 (29.4)	1.12 (0.78–1.60, *p* = 0.542)	1.20 (0.82–1.75, *p* = 0.352)
Grade	Grade I	14 (4.7)	-	-
	Grade II	129 (43.6)	0.81 (0.46–1.45, *p* = 0.486)	0.65 (0.35–1.19, *p* = 0.162)
	Grade III	131 (44.3)	0.97 (0.54–1.72, *p* = 0.907)	1.07 (0.58–1.97, *p* = 0.822)
	Grade IV	22 (7.4)	1.44 (0.72–2.89, *p* = 0.301)	1.81 (0.87–3.77, *p* = 0.111)
Disease	Colon	240 (81.1)	-	-
	Rectum	56 (18.9)	0.93 (0.69–1.26, *p* = 0.644)	0.89 (0.62–1.27, *p* = 0.524)
Histology type	Adenocarcinomas	278 (93.9)	-	-
	Non-adenocarcinomas	18 (6.1)	1.51 (0.93–2.45, *p* = 0.092)	0.74 (0.42–1.32, *p* = 0.306)
BM Radiation	None	138 (46.6)	-	-
	WBRT	91 (30.7)	0.75 (0.57–0.99, *p* = 0.039)	0.70 (0.51–0.95, *p* = 0.022)
	SRS	67 (22.6)	0.49 (0.36–0.68, *p* < 0.001)	0.47 (0.32–0.69, *p* < 0.001)
Systemic therapy	No	141 (47.6)	-	-
	Yes	155 (52.4)	0.38 (0.30–0.49, *p* < 0.001)	0.42 (0.31–0.57, *p* < 0.001)
Surgery of Primary Site	None	77 (26.0)	-	-
	Yes	219 (74.0)	0.53 (0.40–0.70, *p* < 0.001)	0.37 (0.26–0.52, *p* < 0.001)
Year	2010–2015	186 (62.8)	-	-
	2016–2020	110 (37.2)	1.01 (0.78–1.30, *p* = 0.964)	1.01 (0.75–1.36, *p* = 0.938)

Abbreviations: HR: hazard ratio; CI: confidence interval; WBRT: whole-brain radiotherapy; SRS: stereotactic radiosurgery; Systemic: systemic therapy; CDI: Charlson–Deyo Index; HS: high school; Metro: metropolitan; Non-metro: non-metropolitan; Academic/Non-academic: type of treatment facility; Surgery: surgical resection (Yes/No); Year: year of diagnosis (2010–2015 vs. 2016–2020).

**Table 6:** Median survival and survival rates by treatment.

Treatment	N	Median (Months)	3-Month Survival Rate (%)	6-Month Survival Rate (%)	1-Year Survival Rate (%)	2-Year Survival Rate (%)	3-Year Survival Rate (%)
**All**	296	7.82 (5.82–9.66)	72.5 (67.6–77.8)	54.5 (49.0–60.5)	37.0 (31.9–42.9)	20.7 (16.5–25.9)	16.0 (12.3–20.9)
**SRS+Sys**	49	23.26 (17.51–41.95)	98.0 (94.1–100.0)	85.7 (76.5–96.1)	67.3 (55.4–81.8)	46.9 (34.8–63.2)	35.8 (24.4–52.4)
**WBRT+Sys**	51	10.61 (8.48–17.28)	94.1 (87.9–100.0)	74.5 (63.5–87.5)	47.1 (35.2–63.0)	24.5 (14.9–40.0)	17.8 (9.6–32.8)
**Sys**	55	13.83 (10.35–17.12)	92.6 (85.9–99.8)	77.8 (67.5–89.7)	51.9 (40.1–67.1)	22.2 (13.5–36.6)	14.8 (7.8–28.1)
**SRS**	18	3.55 (2.53–13.47)	66.7 (48.1–92.4)	33.3 (17.3–64.1)	20.0 (7.6–52.7)	6.7 (1.0–43.3)	6.7 (1.0–43.3)
**WBRT**	40	3.19 (2.69–7.92)	55.0 (41.6–72.8)	40.0 (27.4–58.5)	20.0 (10.8–37.2)	15.0 (7.2–31.4)	15.0 (7.2–31.4)
**None**	83	2.43 (2.10–3.09)	40.3 (31.0–52.5)	19.6 (12.6–30.3)	14.7 (8.7–24.7)	7.3 (3.4–15.8)	6.1 (2.6–14.3)

Abbreviations: SRS: stereotactic radiosurgery; WBRT: whole-brain radiotherapy; Sys: systemic therapy; SRS+Sys: stereotactic radiosurgery combined with systemic therapy; WBRT+Sys: whole-brain radiotherapy combined with systemic therapy; None: no recorded cancer-directed therapy.

**Table 7:** Median survival and survival rates by metastatic status.

Cancer Type	N	Median (Months)	3-Month Survival Rate (%)	6-Month Survival Rate (%)	1-Year Survival Rate (%)	2-Year Survival Rate (%)	3-Year Survival Rate (%)
**All**	794	6.08 (5.29–6.97)	69.1 (65.9–72.4)	50.0 (46.7–53.7)	33.6 (30.4–37.0)	16.1 (13.7–19.0)	10.4 (8.4–12.9)
**Brain metastasis only**	296	7.82 (5.82–9.66)	72.5 (67.6–77.8)	54.5 (49.0–60.5)	37.0 (31.9–42.9)	20.7 (16.5–25.9)	16.0 (12.3–20.9)
**With other metastases**	498	5.29 (4.53–6.41)	67.0 (63.0–71.3)	47.4 (43.2–52.0)	31.5 (27.7–35.9)	13.4 (10.6–16.8)	7.0 (5.0–9.8)

Abbreviations: N: Number of patients; OS: overall Survival; CI: confidence interval; Colon: colon primary tumor site; Rectum: rectal primary tumor site; Median (months): median survival in months; 3-month/6-month/1-year/2-year/3-year survival rate (%): proportion of patients alive at each respective time point.

## Data Availability

The data supporting the findings of this study are available from the corresponding author upon reasonable request.

## Supplementary Materials

The following are available online at https://www.mdpi.com/article/10.3390/cancers17152531/s1, Figure S1: OR Plot for Full Model with Consolidated Treatment; Figure S2: OR Plot for Reduced Model (Separate and/or Consolidated Treatment); Figure S3: Assessment of the Proportional Hazards Assumption for Age Group; Figure S4: Assessment of the Proportional Hazards Assumption for Charlson-Deyo Comorbidity Index; Figure S5: Assessment of the Proportional Hazards Assumption for Ethnicity; Figure S6: Assessment of the Proportional Hazards Assumption for Histology Type; Figure S7: Assessment of the Proportional Hazards Assumption for Insurance Type; Figure S8: Assessment of the Proportional Hazards Assumption for Race; Figure S9: Assessment of the Proportional Hazards Assumption for Radiation Therapy; Figure S10: Assessment of the Proportional Hazards Assumption for Surgery; Figure S11: Assessment of the Proportional Hazards Assumption for Systemic Therapy; Figure S12: Assessment of the Proportional Hazards Assumption for Composite Treatment Category; Figure S13: Assessment of the Proportional Hazards Assumption for Tumor Size; Figure S14: Assessment of the Proportional Hazards Assumption for Year of Diagnosis; Figure S15: Assessment of the Proportional Hazards Assumption for Year of Diagnosis; Table S1: ICD-O-3 and National Cancer Database Codes Used; Table S2: Multivariable Logistic Regression of Survival Status Among Patients with Colorectal Brain Metastases: Full Model with Individual Treatment Modalities; Table S3: Multivariable Logistic Regression of Survival Status with Consolidated Treatment Modality as a Single Predictor Variable; Table S4: Reduced Multivariable Logistic Regression Model of Survival Status Including Key Clinical and Treatment Predictors (Separate Treatment Variables); Table S5: Reduced Multivariable Logistic Regression Model of Survival Status with Consolidated Treatment Category as a Predictor; Table S6: Full Model of Cox Proportional Hazards Analysis with Treatment as One Variable; Table S7: Reduced Model of the Cox Proportional Hazards Analysis with Separate Treatment Variables Stratified by Education, Grade, and Systemic Treatment Receipt; Table S8: Reduced Model of the Cox Proportional Hazards Analysis with Treatment as One Variable Stratified by Education and Grade; Table S9: Cox Proportional Hazards Full Model 1, Stratified by Sex, Education, Tumor Grade, Histology, and Systemic Therapy; Table S10: Cox Proportional Hazards Full Model 2, Stratified by Sex, Education Level, Tumor Grade, Cancer Type, and Treatment Summary; Table S11: Accelerated Failure Time (AFT) Model 1: Full Model with Treatment as Three Separate Variables; Table S12: Accelerated Failure Time (AFT) Model 2: Full Model with Treatment as a Single Summary Variable.

## Abbreviations

The following abbreviations are used in this manuscript:

ACS	American Cancer Society
BM	Brain metastasis
CDS	Charlson–Deyo score
CI	Confidence interval
CNS	Central nervous system
CoC	Commission on Cancer
CRC	Colorectal cancer
HR	Hazard ratio
IQR	Interquartile range
Mri	Magnetic resonance imaging
NCDB	National Cancer Database
OR	Odds ratio
OS	Overall survival
SRS	Stereotactic radiosurgery
SRS+Sys	SRS combined with systemic therapy
STROBE	Strengthening the Reporting of Observational Studies in Epidemiology
Sys	Systemic therapy
US	United States
WBRT	Whole-brain radiotherapy
