# Supplementary material for: Survival in Patients with Colorectal Cancer and Isolated Brain Metastases: Temporal Trends and Prognostic Factors from the National Cancer Database (2010–2020)
Source: Cancers (Basel). 2025 Jul 31;17(15):2531. doi: 10.3390/cancers17152531 (PMC12345898; doi:10.3390/cancers17152531)

List of Supplementary Tables:

Supplementary Table S1: ICD-O-3 and National Cancer Database Codes Used.

Supplementary Table S2: Multivariable Logistic Regression of Survival Status Among Patients with Colorectal Brain Metastases: 6 Months.

Supplementary Table S3: Multivariable Logistic Regression of Survival Status Among Patients with Colorectal Brain Metastases: 12 Months.

Supplementary Table S4: Multivariable Logistic Regression of Survival Status Among Patients with Colorectal Brain Metastases: 18 Months.

Supplementary Table S5: Multivariable Logistic Regression of Survival Status Among Patients with Colorectal Brain Metastases: 24 Months.

Supplementary Table S6. Full Model of Cox Proportional Hazards Analysis with Treatment as One Variable.

Supplementary Table S7. Reduced Model of the Cox Proportional Hazards Analysis with Separate Treatment Variables Stratified by Education, Grade, and Systemic Treatment Receipt.

Supplementary Table S8. Reduced Model of the Cox Proportional Hazards Analysis with Treatment as One Variable Stratified by Education and Grade.

Supplementary Table S9. Cox Proportional Hazards Full Model 1, Stratified by Sex, Education, Tumor Grade, Histology, and Systemic Therapy.

Supplementary Table S10. Cox Proportional Hazards Full Model 2, Stratified by Sex, Education Level, Tumor Grade, Cancer Type, and Treatment Summary.

Supplementary Table S11. Accelerated Failure Time (AFT) Model 1: Full Model with Treatment as Three Separate Variables.

Supplementary Table S12. Accelerated Failure Time (AFT) Model 2: Full Model with Treatment as a Single Summary Variable.

List of Supplementary Figures:

Supplementary Figure S1: OR Plot with Time Cutoff at 12 Months.

Supplementary Figure S2: OR Plot with Time Cutoff at 18 Months.

Supplementary Figure S3: OR Plot with Time Cutoff at 24 Months.

Supplementary Figure S4: Assessment of the Proportional Hazards Assumption for Age Group.

Supplementary Figure S5: Assessment of the Proportional Hazards Assumption for Charlson-Deyo Comorbidity Index

Supplementary Figure S6. Assessment of the Proportional Hazards Assumption for Ethnicity.

Supplementary Figure S7. Assessment of the Proportional Hazards Assumption for Histology Type.

Supplementary Figure S8. Assessment of the Proportional Hazards Assumption for Insurance Type.

Supplementary Figure S9. Assessment of the Proportional Hazards Assumption for Race.

Supplementary Figure S10. Assessment of the Proportional Hazards Assumption for Radiation Therapy

Supplementary Figure S11. Assessment of the Proportional Hazards Assumption for Surgery.

Supplementary Figure S12. Assessment of the Proportional Hazards Assumption for Systemic Therapy.

Supplementary Figure S13. Assessment of the Proportional Hazards Assumption for Composite Treatment Category

Supplementary Figure S14. Assessment of the Proportional Hazards Assumption for Tumor Size.

Supplementary Figure S15. Assessment of the Proportional Hazards Assumption for Year of Diagnosis.

Supplementary Table S1: ICD-O-3 and National Cancer Database Codes Used.

**Category**	**ICD-O-3 Code**	**Description**
	**Topography Codes**	
Colon	C18.0	Cecum
Colon	C18.1	Appendix
Colon	C18.2	Ascending colon
Colon	C18.3	Hepatic flexure of colon
Colon	C18.4	Transverse colon
Colon	C18.5	Splenic flexure of colon
Colon	C18.6	Descending colon
Colon	C18.7	Sigmoid colon
Colon	C18.8	Overlapping lesion of colon
Colon	C18.9	Colon, NOS
Rectum	C19.9	Rectosigmoid junction
Rectum	C20.9	Rectum, NOS
	**Morphology Codes**	
Adenocarcinoma	8140	Adenocarcinoma, NOS
Adenocarcinoma	8210	Adenocarcinoma in adenomatous polyp
Adenocarcinoma	8255	Adenocarcinoma with mixed subtypes
Adenocarcinoma	8261	Mucinous adenocarcinoma
Adenocarcinoma	8263	Adenocarcinoma in villous adenoma
Adenocarcinoma	8480	Mucinous adenocarcinoma (colloid)
Adenocarcinoma	8490	Signet ring cell carcinoma
Neuroendocrine	8013	Large cell neuroendocrine carcinoma

Neuroendocrine	8041	Small cell carcinoma, NOS
Neuroendocrine	8240	Carcinoid tumor, NOS
Neuroendocrine	8244	Mixed adenoneuroendocrine carcinoma
Neuroendocrine	8246	Neuroendocrine carcinoma, NOS
Neuroendocrine	8249	Atypical carcinoid tumor
Neuroendocrine	8574	Adenocarcinoma with neuroendocrine differentiation
Squamous/Adenosquamous	8070	Squamous cell carcinoma, NOS
Squamous/Adenosquamous	8072	Squamous cell carcinoma, large cell, nonkeratinizing
Squamous/Adenosquamous	8083	Basaloid squamous cell carcinoma
Squamous/Adenosquamous	8560	Adenosquamous carcinoma
Poorly/Undifferentiated	8000	Neoplasm, malignant
Poorly/Undifferentiated	8010	Carcinoma, NOS
Poorly/Undifferentiated	8020	Undifferentiated carcinoma, NOS

National Cancer Database – Grouping and Coding

Category	Code	Description	Variable Name	Site/Context
Chemotherapy	01, 02, 03	Chemotherapy administered	RX_SUMM_CHEMO	First course treatment
	00, 82, 85, 86, 87, 88, 99	Chemotherapy not administered or unknown		
Immunotherapy	01	Immunotherapy administered	RX_SUMM_IMMUNOTHERAPY	
	00, 82, 85, 86, 87, 88, 99	Immunotherapy not administered or unknown		
Surgery	0, 99	No surgery or unknown	RX_SUMM_SURG_PRIM_SITE	Primary site surgery

	10–27	Local excision (tumor destruction, minimal surgery)		
	30–90	Definitive surgery (more extensive resection)		
Radiation*	0	No radiation to whole brain or SRS	PHASE_I_RT_VOLUME	Radiation Phase I volume code
	12	Whole Brain Radiation Therapy (WBRT)		Radiation to brain
	13	Stereotactic Radiosurgery (SRS)		
Systemic Treatment	1	Either chemotherapy or immunotherapy (or both) administered	systemic_yn	Any systemic (chemo or immuno)
	0	Neither therapy administered		
Final Treatment Groupings	SRS+Sys	SRS + systemic therapy	TX_SUMM	Brain metastasis treatment grouping
	SRS	SRS only, no systemic		
	WBRT+Sys	WBRT + systemic therapy		
	WBRT	WBRT only, no systemic		
	Sys	Systemic therapy only, no radiation		
	None	Neither radiation nor systemic therapy		

* Brain-directed radiation only; primary site radiation not documented.

Abbreviations: SRS: Stereotactic Radiosurgery; SRS+Sys: Stereotactic Radiosurgery + systemic therapy; Sys: Systemic therapy; WBRT: Whole Brain Radiation Therapy; WBRT+Sys: Whole Brain Radiation Therapy + systemic therapy

Supplementary Table S2: Multivariable Logistic Regression of Survival Status Among Patients with Colorectal Brain Metastases: 6 Months.

This table shows univariable and multivariable logistic regression analyses evaluating predictors of 6-month survival among patients with brain-only colorectal metastases. No sociodemographic variable, including age, sex, race, ethnicity, income, region, education level, or insurance type, was independently associated with 6-month survival after adjustment. Although older age, public insurance, and lower education levels yielded lower survival in univariable analyses, these were not statistically significant in the adjusted model. Clinical factors such as tumor size, grade, comorbidity (Charlson-Deyo), and primary tumor site were also not significantly associated with 6-month survival, except for high-grade tumors (Grade IV), which were linked to higher odds of death. SRS, systemic therapy, WBRT, and surgical resection were all significantly associated with higher odds of survival.

Variable	Group	Alive	Dead	OR (univariable)	OR (multivariable)
**Age group**	40-64 years	90 (73.2)	33 (26.8)	-	-
	65-90 years	72 (41.6)	101 (58.4)	3.83 (2.34-6.37, p<0.001)	1.47 (0.61-3.53, p=0.389)
**Gender**	Male	87 (62.1)	53 (37.9)	-	-
	Female	75 (48.1)	81 (51.9)	1.77 (1.12-2.83, p=0.016)	1.37 (0.72-2.62, p=0.336)
**Race**	White	138 (53.3)	121 (46.7)	-	-
	Non-white	24 (64.9)	13 (35.1)	0.62 (0.29-1.25, p=0.188)	0.38 (0.13-1.04, p=0.065)
**Ethnicity**	Non-Hispanic	149 (55.6)	119 (44.4)	-	-
	Hispanic	13 (46.4)	15 (53.6)	1.44 (0.66-3.20, p=0.356)	1.45 (0.50-4.30, p=0.495)
**Facility type**	Academic	51 (56.7)	39 (43.3)	-	-
	Nonacademic	111 (53.9)	95 (46.1)	1.12 (0.68-1.85, p=0.658)	0.97 (0.45-2.07, p=0.928)
**Median income**	<$74,063	101 (55.5)	81 (44.5)	-	-
	≥$74,063	61 (53.5)	53 (46.5)	1.08 (0.68-1.73, p=0.738)	1.18 (0.51-2.76, p=0.706)
**Insurance type**	Private	61 (67.8)	29 (32.2)	-	-
	Public	83 (44.6)	103 (55.4)	2.61 (1.55-4.47, p<0.001)	1.02 (0.39-2.59, p=0.974)
	Uninsured	18 (90.0)	2 (10.0)	0.23 (0.04-0.88, p=0.062)	0.14 (0.02-0.81, p=0.047)
**Rate of no high school completion**	≥9.1%	90 (57.0)	68 (43.0)	-	-
	<9.1%	72 (52.2)	66 (47.8)	1.21 (0.77-1.92, p=0.409)	1.36 (0.61-3.05, p=0.451)

**Region**	Metropolitan	127 (54.7)	105 (45.3)	-	-
	Non-metropolitan	35 (54.7)	29 (45.3)	1.00 (0.57-1.75, p=0.994)	1.11 (0.48-2.60, p=0.803)
**Charlson-Deyo index**	0	133 (60.5)	87 (39.5)	-	-
	1	19 (44.2)	24 (55.8)	1.93 (1.00-3.77, p=0.051)	2.12 (0.88-5.22, p=0.097)
	2-3	10 (30.3)	23 (69.7)	3.52 (1.64-8.07, p=0.002)	4.24 (1.49-12.88, p=0.008)
**Tumor size**	<40 mm	31 (53.4)	27 (46.6)	-	-
	40-70 mm	85 (56.3)	66 (43.7)	0.89 (0.49-1.64, p=0.711)	1.45 (0.59-3.68, p=0.426)
	>70 mm	46 (52.9)	41 (47.1)	1.02 (0.53-2.00, p=0.946)	2.44 (0.91-6.87, p=0.082)
**Grade**	Grade I	10 (71.4)	4 (28.6)	-	-
	Grade II	75 (58.1)	54 (41.9)	1.80 (0.57-6.84, p=0.342)	1.84 (0.33-10.51, p=0.482)
	Grade III	70 (53.4)	61 (46.6)	2.18 (0.69-8.26, p=0.207)	4.14 (0.74-24.54, p=0.108)
	Grade IV	7 (31.8)	15 (68.2)	5.36 (1.31-25.71, p=0.025)	12.43 (1.61-104.15, p=0.017)
**Disease Site**	Colon	121 (50.4)	119 (49.6)	-	-
	Rectum	41 (73.2)	15 (26.8)	0.37 (0.19-0.69, p=0.003)	0.20 (0.07-0.53, p=0.002)
**Radiation**	None	60 (43.5)	78 (56.5)	-	-
	WBRT	54 (59.3)	37 (40.7)	0.53 (0.31-0.90, p=0.019)	0.43 (0.20-0.92, p=0.032)
	SRS	48 (71.6)	19 (28.4)	0.30 (0.16-0.56, p<0.001)	0.30 (0.12-0.70, p=0.006)
**Systemic**	No	39 (27.7)	102 (72.3)	-	-
	Yes	123 (79.4)	32 (20.6)	0.10 (0.06-0.17, p<0.001)	0.10 (0.05-0.20, p<0.001)
**Surgery**	None	31 (40.3)	46 (59.7)	-	-
	Yes	131 (59.8)	88 (40.2)	0.45 (0.26-0.77, p=0.003)	0.14 (0.05-0.33, p<0.001)
**Year of Diagnosis**	2010-2015	103 (55.4)	83 (44.6)	-	-
	2016-2020	59 (53.6)	51 (46.4)	1.07 (0.67-1.72, p=0.771)	0.91 (0.45-1.82, p=0.785)

Abbreviations: OR: Odds Ratio; p: p-value; SRS: Stereotactic Radiosurgery; WBRT: Whole Brain Radiation Therapy; Charlson-Deyo index: Comorbidity scoring index.

Supplementary Table S3: Multivariable Logistic Regression of Survival Status Among Patients with Colorectal Brain Metastases: 12 Months.

This table shows univariable and multivariable logistic regression analyses evaluating predictors of 12-month survival among patients with brain-only colorectal metastases. No sociodemographic variable, including age, sex, race, ethnicity, income, region, education level, or insurance type, was independently associated with 12-month survival after adjustment. Although older age, public insurance, and lower education levels were associated with lower survival in univariable models, these associations were not statistically significant in the multivariable analysis. Clinical factors such as tumor grade, comorbidity (Charlson-Deyo), and primary tumor site also showed no significant association with 12-month survival, except for large tumors (>70 mm), which were associated with higher odds of death. SRS, systemic therapy, and surgical resection were significantly associated with higher odds of survival.

Variable	Group	Alive	Dead	OR (univariable)	OR (multivariable)
**Age group**	40-64 years	64 (52.0)	59 (48.0)	-	-
	65-90 years	47 (27.2)	126 (72.8)	2.91 (1.79-4.76, p<0.001)	1.26 (0.56-2.81, p=0.572)
**Gender**	Male	60 (42.9)	80 (57.1)	-	-
	Female	51 (32.7)	105 (67.3)	1.54 (0.96-2.48, p=0.072)	1.30 (0.72-2.35, p=0.385)
**Race**	White	97 (37.5)	162 (62.5)	-	-
	Non-white	14 (37.8)	23 (62.2)	0.98 (0.49-2.04, p=0.964)	0.96 (0.39-2.40, p=0.921)
**Ethnicity**	Non-Hispanic	99 (36.9)	169 (63.1)	-	-
	Hispanic	12 (42.9)	16 (57.1)	0.78 (0.36-1.75, p=0.539)	0.47 (0.18-1.26, p=0.129)
**Facility type**	Academic	34 (37.8)	56 (62.2)	-	-
	Nonacademic	77 (37.4)	129 (62.6)	1.02 (0.61-1.69, p=0.948)	0.88 (0.45-1.71, p=0.702)
**Median income**	<$74,063	69 (37.9)	113 (62.1)	-	-
	≥$74,063	42 (36.8)	72 (63.2)	1.05 (0.65-1.70, p=0.853)	0.97 (0.44-2.14, p=0.941)
**Insurance type**	Private	43 (47.8)	47 (52.2)	-	-
	Public	54 (29.0)	132 (71.0)	2.24 (1.33-3.78, p=0.002)	1.31 (0.56-3.04, p=0.535)
	Uninsured	14 (70.0)	6 (30.0)	0.39 (0.13-1.07, p=0.078)	0.31 (0.08-1.05, p=0.068)
	≥9.1%	61 (38.6)	97 (61.4)	-	-

**Rate of No high school completion**	<9.1%	50 (36.2)	88 (63.8)	1.11 (0.69-1.78, p=0.674)	1.16 (0.56-2.42, p=0.698)
**Region**	Metropolitan	87 (37.5)	145 (62.5)	-	-
	Non-metropolitan	24 (37.5)	40 (62.5)	1.00 (0.57-1.79, p=1.000)	0.95 (0.45-2.06, p=0.899)
**Charlson-Deyo index**	0	90 (40.9)	130 (59.1)	-	-
	1	14 (32.6)	29 (67.4)	1.43 (0.73-2.94, p=0.307)	1.13 (0.50-2.64, p=0.771)
	2-3	7 (21.2)	26 (78.8)	2.57 (1.12-6.66, p=0.035)	2.13 (0.78-6.36, p=0.154)
**Tumor size**	<40 mm	24 (41.4)	34 (58.6)	-	-
	40-70 mm	58 (38.4)	93 (61.6)	1.13 (0.61-2.09, p=0.694)	1.40 (0.63-3.14, p=0.410)
	>70 mm	29 (33.3)	58 (66.7)	1.41 (0.71-2.81, p=0.325)	2.57 (1.06-6.35, p=0.038)
**Grade**	Grade I	7 (50.0)	7 (50.0)	-	-
	Grade II	53 (41.1)	76 (58.9)	1.43 (0.47-4.42, p=0.523)	0.79 (0.19-3.23, p=0.742)
	Grade III	46 (35.1)	85 (64.9)	1.85 (0.60-5.71, p=0.277)	1.59 (0.39-6.56, p=0.515)
	Grade IV	5 (22.7)	17 (77.3)	3.40 (0.82-15.38, p=0.097)	3.28 (0.57-20.38, p=0.190)
**Disease Site**	Colon	86 (35.8)	154 (64.2)	-	-
	Rectum	25 (44.6)	31 (55.4)	0.69 (0.38-1.26, p=0.222)	0.68 (0.30-1.54, p=0.357)
**Radiation**	None	42 (30.4)	96 (69.6)	-	-
	WBRT	32 (35.2)	59 (64.8)	0.81 (0.46-1.42, p=0.454)	0.94 (0.46-1.90, p=0.852)
	SRS	37 (55.2)	30 (44.8)	0.35 (0.19-0.65, p=0.001)	0.41 (0.19-0.87, p=0.022)
**Systemic**	No	25 (17.7)	116 (82.3)	-	-
	Yes	86 (55.5)	69 (44.5)	0.17 (0.10-0.29, p<0.001)	0.22 (0.11-0.42, p<0.001)
**Surgery**	None	16 (20.8)	61 (79.2)	-	-
	Yes	95 (43.4)	124 (56.6)	0.34 (0.18-0.62, p=0.001)	0.15 (0.06-0.33, p<0.001)
**Year of Diagnosis**	2010-2015	70 (37.6)	116 (62.4)	-	-
	2016-2020	41 (37.3)	69 (62.7)	1.02 (0.63-1.66, p=0.950)	0.87 (0.46-1.65, p=0.673)

Abbreviations: OR: Odds Ratio; p: p-value; SRS: Stereotactic Radiosurgery; WBRT: Whole Brain Radiation Therapy; Charlson-Deyo index: Comorbidity scoring index.

Supplementary Table S4: Multivariable Logistic Regression of Survival Status Among Patients with Colorectal Brain Metastases: 18 Months.

This table shows univariable and multivariable logistic regression analyses evaluating predictors of 18-month survival among patients with brain-only colorectal metastases. No sociodemographic variable, including age, sex, race, ethnicity, income, region, education level, or insurance type, was independently associated with 18-month survival after adjustment. Clinical factors such as comorbidity, tumor grade, and primary tumor site were not significantly associated with 18-month survival. However, larger tumor size (>40 mm) was associated with higher odds of death. SRS, systemic therapy, and surgical resection were all significantly associated with higher odds of survival.

Variable	Group	Alive	Dead	OR (univariable)	OR (multivariable)
**Age group**	40-64 years	46 (37.4)	77 (62.6)	-	-
	65-90 years	32 (18.5)	141 (81.5)	2.63 (1.56-4.50, p<0.001)	1.24 (0.51-2.98, p=0.630)
**Gender**	Male	37 (26.4)	103 (73.6)	-	-
	Female	41 (26.3)	115 (73.7)	1.01 (0.60-1.69, p=0.977)	0.74 (0.39-1.42, p=0.371)
**Race**	White	67 (25.9)	192 (74.1)	-	-
	Non-white	11 (29.7)	26 (70.3)	0.82 (0.40-1.82, p=0.618)	0.68 (0.27-1.77, p=0.413)
**Ethnicity**	Non-Hispanic	72 (26.9)	196 (73.1)	-	-
	Hispanic	6 (21.4)	22 (78.6)	1.35 (0.56-3.78, p=0.536)	0.92 (0.31-3.16, p=0.894)
**Facility type**	Academic	25 (27.8)	65 (72.2)	-	-
	Nonacademic	53 (25.7)	153 (74.3)	1.11 (0.63-1.93, p=0.713)	1.05 (0.51-2.13, p=0.889)
**Median income**	<$74,063	50 (27.5)	132 (72.5)	-	-
	≥$74,063	28 (24.6)	86 (75.4)	1.16 (0.68-2.01, p=0.580)	1.06 (0.45-2.54, p=0.894)
**Insurance type**	Private	32 (35.6)	58 (64.4)	-	-
	Public	37 (19.9)	149 (80.1)	2.22 (1.26-3.90, p=0.005)	1.43 (0.58-3.52, p=0.435)
	Uninsured	9 (45.0)	11 (55.0)	0.67 (0.25-1.83, p=0.431)	0.60 (0.18-2.04, p=0.406)
**Rate of no high school completion**	≥9.1%	45 (28.5)	113 (71.5)	-	-
	<9.1%	33 (23.9)	105 (76.1)	1.27 (0.75-2.15, p=0.374)	1.39 (0.63-3.17, p=0.419)

**Region**	Metropolitan	60 (25.9)	172 (74.1)	-	-
	Non-metropolitan	18 (28.1)	46 (71.9)	0.89 (0.49-1.69, p=0.716)	1.04 (0.45-2.43, p=0.934)
**Charlson-Deyo index**	0	63 (28.6)	157 (71.4)	-	-
	1	9 (20.9)	34 (79.1)	1.52 (0.71-3.53, p=0.302)	1.17 (0.47-3.13, p=0.749)
	2-3	6 (18.2)	27 (81.8)	1.81 (0.76-5.02, p=0.214)	1.88 (0.65-6.15, p=0.268)
**Tumor size**	<40 mm	21 (36.2)	37 (63.8)	-	-
	40-70 mm	35 (23.2)	116 (76.8)	1.88 (0.97-3.62, p=0.059)	2.76 (1.20-6.45, p=0.017)
	>70 mm	22 (25.3)	65 (74.7)	1.68 (0.81-3.46, p=0.160)	3.30 (1.34-8.40, p=0.010)
**Grade**	Grade I	3 (21.4)	11 (78.6)	-	-
	Grade II	38 (29.5)	91 (70.5)	0.65 (0.14-2.23, p=0.531)	0.35 (0.07-1.50, p=0.183)
	Grade III	34 (26.0)	97 (74.0)	0.78 (0.17-2.67, p=0.713)	0.67 (0.12-2.89, p=0.614)
	Grade IV	3 (13.6)	19 (86.4)	1.73 (0.28-10.82, p=0.544)	1.62 (0.21-13.00, p=0.638)
**Disease Site**	Colon	61 (25.4)	179 (74.6)	-	-
	Rectum	17 (30.4)	39 (69.6)	0.78 (0.42-1.51, p=0.451)	0.58 (0.24-1.41, p=0.224)
**Radiation**	None	25 (18.1)	113 (81.9)	-	-
	WBRT	22 (24.2)	69 (75.8)	0.69 (0.36-1.33, p=0.268)	0.58 (0.26-1.28, p=0.180)
	SRS	31 (46.3)	36 (53.7)	0.26 (0.13-0.49, p<0.001)	0.26 (0.12-0.59, p=0.001)
**Systemic**	No	17 (12.1)	124 (87.9)	-	-
	Yes	61 (39.4)	94 (60.6)	0.21 (0.11-0.38, p<0.001)	0.31 (0.15-0.63, p=0.002)
**Surgery**	None	8 (10.4)	69 (89.6)	-	-
	Yes	70 (32.0)	149 (68.0)	0.25 (0.10-0.51, p<0.001)	0.11 (0.04-0.28, p<0.001)
**Year of Diagnosis**	2010-2015	47 (25.3)	139 (74.7)	-	-
	2016-2020	31 (28.2)	79 (71.8)	0.86 (0.51-1.47, p=0.583)	0.63 (0.32-1.27, p=0.198)

Abbreviations: OR: Odds Ratio; p: p-value; SRS: Stereotactic Radiosurgery; WBRT: Whole Brain Radiation Therapy; Charlson-Deyo index: Comorbidity scoring index.

Supplementary Table S5: Multivariable Logistic Regression of Survival Status Among Patients with Colorectal Brain Metastases: 24 Months.

This table shows univariable and multivariable logistic regression analyses evaluating predictors of 24-month survival among patients with brain-only colorectal metastases. No sociodemographic variable, including age, sex, race, ethnicity, income, region, education level, or insurance type, was independently associated with 24-month survival after adjustment. Clinical factors such as comorbidity, tumor grade, and primary tumor site were not significantly associated with survival. However, larger tumor size (>40 mm) was associated with higher odds of death. SRS, systemic therapy, and surgical resection remained significantly associated with higher odds of survival.

Variable	Group	Alive	Dead	OR (univariable)	OR (multivariable)
**Age group**	40-64 years	46 (37.4)	77 (62.6)	-	-
	65-90 years	32 (18.5)	141 (81.5)	2.63 (1.56-4.50, p<0.001)	1.24 (0.51-2.98, p=0.630)
**Gender**	Male	37 (26.4)	103 (73.6)	-	-
	Female	41 (26.3)	115 (73.7)	1.01 (0.60-1.69, p=0.977)	0.74 (0.39-1.42, p=0.371)
**Race**	White	67 (25.9)	192 (74.1)	-	-
	Non-white	11 (29.7)	26 (70.3)	0.82 (0.40-1.82, p=0.618)	0.68 (0.27-1.77, p=0.413)
**Ethnicity**	Non-Hispanic	72 (26.9)	196 (73.1)	-	-
	Hispanic	6 (21.4)	22 (78.6)	1.35 (0.56-3.78, p=0.536)	0.92 (0.31-3.16, p=0.894)
**Facility type**	Academic	25 (27.8)	65 (72.2)	-	-
	Nonacademic	53 (25.7)	153 (74.3)	1.11 (0.63-1.93, p=0.713)	1.05 (0.51-2.13, p=0.889)
**Median income**	<$74,063	50 (27.5)	132 (72.5)	-	-
	≥$74,063	28 (24.6)	86 (75.4)	1.16 (0.68-2.01, p=0.580)	1.06 (0.45-2.54, p=0.894)
**Insurance type**	Private	32 (35.6)	58 (64.4)	-	-
	Public	37 (19.9)	149 (80.1)	2.22 (1.26-3.90, p=0.005)	1.43 (0.58-3.52, p=0.435)
	Uninsured	9 (45.0)	11 (55.0)	0.67 (0.25-1.83, p=0.431)	0.60 (0.18-2.04, p=0.406)
**Rate of no high school completion**	≥9.1%	45 (28.5)	113 (71.5)	-	-
	<9.1%	33 (23.9)	105 (76.1)	1.27 (0.75-2.15, p=0.374)	1.39 (0.63-3.17, p=0.419)

**Region**	Metropolitan	60 (25.9)	172 (74.1)	-	-
	Non-metropolitan	18 (28.1)	46 (71.9)	0.89 (0.49-1.69, p=0.716)	1.04 (0.45-2.43, p=0.934)
**Charlson-Deyo index**	0	63 (28.6)	157 (71.4)	-	-
	1	9 (20.9)	34 (79.1)	1.52 (0.71-3.53, p=0.302)	1.17 (0.47-3.13, p=0.749)
	2-3	6 (18.2)	27 (81.8)	1.81 (0.76-5.02, p=0.214)	1.88 (0.65-6.15, p=0.268)
**Tumor size**	<40 mm	21 (36.2)	37 (63.8)	-	-
	40-70 mm	35 (23.2)	116 (76.8)	1.88 (0.97-3.62, p=0.059)	2.76 (1.20-6.45, p=0.017)
	>70 mm	22 (25.3)	65 (74.7)	1.68 (0.81-3.46, p=0.160)	3.30 (1.34-8.40, p=0.010)
**Grade**	Grade I	3 (21.4)	11 (78.6)	-	-
	Grade II	38 (29.5)	91 (70.5)	0.65 (0.14-2.23, p=0.531)	0.35 (0.07-1.50, p=0.183)
	Grade III	34 (26.0)	97 (74.0)	0.78 (0.17-2.67, p=0.713)	0.67 (0.12-2.89, p=0.614)
	Grade IV	3 (13.6)	19 (86.4)	1.73 (0.28-10.82, p=0.544)	1.62 (0.21-13.00, p=0.638)
**Disease Site**	Colon	61 (25.4)	179 (74.6)	-	-
	Rectum	17 (30.4)	39 (69.6)	0.78 (0.42-1.51, p=0.451)	0.58 (0.24-1.41, p=0.224)
**Radiation**	None	25 (18.1)	113 (81.9)	-	-
	WBRT	22 (24.2)	69 (75.8)	0.69 (0.36-1.33, p=0.268)	0.58 (0.26-1.28, p=0.180)
	SRS	31 (46.3)	36 (53.7)	0.26 (0.13-0.49, p<0.001)	0.26 (0.12-0.59, p=0.001)
**Systemic**	No	17 (12.1)	124 (87.9)	-	-
	Yes	61 (39.4)	94 (60.6)	0.21 (0.11-0.38, p<0.001)	0.31 (0.15-0.63, p=0.002)
**Surgery**	None	8 (10.4)	69 (89.6)	-	-
	Yes	70 (32.0)	149 (68.0)	0.25 (0.10-0.51, p<0.001)	0.11 (0.04-0.28, p<0.001)
**Year of Diagnosis**	2010-2015	47 (25.3)	139 (74.7)	-	-
	2016-2020	31 (28.2)	79 (71.8)	0.86 (0.51-1.47, p=0.583)	0.63 (0.32-1.27, p=0.198)

Abbreviations: OR: Odds Ratio; p: p-value; SRS: Stereotactic Radiosurgery; WBRT: Whole Brain Radiation Therapy; Charlson-Deyo index: Comorbidity scoring index.

Supplementary Table S6. Full Model of Cox Proportional Hazards Analysis with Treatment as One Variable.

This table presents a multivariable Cox regression model evaluating overall survival in patients with brain-only colorectal metastases, incorporating demographic, clinical, socioeconomic, and treatment-related factors. Treatment was modeled as a single categorical variable capturing distinct modality combinations. Several treatment categories, particularly those involving radiation or systemic therapy alone, were significantly associated with worse survival relative to the SRS+systemic reference group. Among clinical and non-treatment variables, older age and higher Charlson-Deyo comorbidity scores were independently associated with poorer survival. Lower high school completion rates also showed a modest association with decreased survival. No consistent differences in survival were observed by race, ethnicity, region, income, or tumor grade after adjustment.

Variable	Group	N (%)	HR (univariable)	HR (multivariable)
Age group	40-64 years	123 (41.6)	-	-
	65-90 years	173 (58.4)	1.98 (1.54-2.55, p<0.001)	1.43 (1.00-2.03, p=0.048)
Gender	Male	140 (47.3)	-	-
	Female	156 (52.7)	1.09 (0.86-1.39, p=0.484)	1.03 (0.79-1.33, p=0.845)
Race	White	259 (87.5)	-	-
	Non-white	37 (12.5)	0.81 (0.55-1.18, p=0.272)	0.67 (0.44-1.02, p=0.062)
Ethnicity	Non-Hispanic	268 (90.5)	-	-
	Hispanic	28 (9.5)	0.99 (0.65-1.49, p=0.945)	0.98 (0.62-1.54, p=0.915)
Facility type	Academic	90 (30.4)	-	-
	Nonacademic	206 (69.6)	1.09 (0.84-1.41, p=0.530)	0.93 (0.69-1.24, p=0.611)
Median income	<$74,063	182 (61.5)	-	-
	≥$74,063	114 (38.5)	1.10 (0.86-1.41, p=0.453)	0.81 (0.55-1.19, p=0.274)
Insurance type	Private	90 (30.4)	-	-
	Public	186 (62.8)	1.72 (1.30-2.26, p<0.001)	1.03 (0.70-1.51, p=0.883)
	Uninsured	20 (6.8)	0.71 (0.41-1.24, p=0.234)	0.69 (0.38-1.23, p=0.208)
Rate of No high school	≥9.1%	158 (53.4)	-	-
	<9.1%	138 (46.6)	1.26 (0.99-1.61, p=0.065)	1.45 (1.02-2.06, p=0.039)

Region	Metropolitan	232 (78.4)	-	-
	Non-metropolitan	64 (21.6)	0.95 (0.71-1.28, p=0.748)	0.99 (0.70-1.42, p=0.978)
Charlson-Deyo index	0	220 (74.3)	-	-
	1	43 (14.5)	1.32 (0.94-1.86, p=0.111)	1.51 (1.05-2.16, p=0.026)
	2-3	33 (11.1)	1.54 (1.04-2.26, p=0.029)	1.36 (0.89-2.08, p=0.153)
Tumor size	<40 mm	58 (19.6)	-	-
	40-70 mm	151 (51.0)	1.10 (0.79-1.52, p=0.585)	0.90 (0.63-1.28, p=0.552)
	>70 mm	87 (29.4)	1.12 (0.78-1.60, p=0.542)	0.98 (0.67-1.43, p=0.928)
Grade	Grade I	14 (4.7)	-	-
	Grade II	129 (43.6)	0.81 (0.46-1.45, p=0.486)	0.85 (0.47-1.54, p=0.588)
	Grade III	131 (44.3)	0.97 (0.54-1.72, p=0.907)	1.19 (0.65-2.20, p=0.573)
	Grade IV	22 (7.4)	1.44 (0.72-2.89, p=0.301)	1.74 (0.83-3.64, p=0.140)
Disease	Colon	240 (81.1)	-	-
	Rectum	56 (18.9)	0.93 (0.69-1.26, p=0.644)	1.11 (0.78-1.57, p=0.568)
Histology type	Adenocarcinomas	278 (93.9)	-	-
	Non-adenocarcinomas	18 (6.1)	1.51 (0.93-2.45, p=0.092)	1.01 (0.57-1.78, p=0.972)
Treatment	SRS+Sys	49 (16.6)	-	-
	WBRT+Sys	51 (17.2)	1.67 (1.07-2.61, p=0.023)	1.98 (1.22-3.22, p=0.005)
	Sys	55 (18.6)	1.68 (1.09-2.59, p=0.019)	2.23 (1.38-3.59, p=0.001)
	SRS	18 (6.1)	3.79 (2.11-6.81, p<0.001)	3.78 (1.97-7.23, p<0.001)
	WBRT	40 (13.5)	2.72 (1.71-4.34, p<0.001)	3.58 (2.11-6.06, p<0.001)
	None	83 (28.0)	4.51 (3.01-6.75, p<0.001)	5.82 (3.67-9.22, p<0.001)
Year	2010-2015	186 (62.8)	-	-
	2016-2020	110 (37.2)	1.01 (0.78-1.30, p=0.964)	1.20 (0.90-1.61, p=0.215)

Abbreviations: HR: Hazard Ratio; p: p-value; SRS: Stereotactic Radiosurgery; SRS+Sys: Stereotactic Radiosurgery + systemic therapy; Sys: Systemic therapy; WBRT: Whole Brain Radiation Therapy; WBRT+Sys: Whole Brain Radiation Therapy + systemic therapy.

Supplementary Table S7. Reduced Model of the Cox Proportional Hazards Analysis with Separate Treatment Variables Stratified by Education, Grade, and Systemic Treatment Receipt.

This model evaluates overall survival using separate radiation and surgery variables, stratified by key clinical factors. Surgical resection and SRS were significantly associated with improved survival. Older age was independently linked to worse outcomes. No adjusted associations were observed for race, facility type, histology, or diagnosis year.

Variable	Group	N (%)	HR (univariable)	HR (multivariable)
Age group	40-64 years	123 (41.6)	-	-
	65-90 years	173 (58.4)	1.98 (1.54-2.55, p<0.001)	1.57 (1.17-2.10, p=0.002)
Race	White	259 (87.5)	-	-
	Non-white	37 (12.5)	0.81 (0.55-1.18, p=0.272)	0.76 (0.50-1.16, p=0.208)
Facility type	Academic	90 (30.4)	-	-
	Nonacademic	206 (69.6)	1.09 (0.84-1.41, p=0.530)	1.24 (0.92-1.67, p=0.159)
Histology type	Adenocarcinomas	278 (93.9)	-	-
	Non-adenocarcinomas	18 (6.1)	1.51 (0.93-2.45, p=0.092)	0.69 (0.39-1.25, p=0.225)
Year	2010-2015	186 (62.8)	-	-
	2016-2020	110 (37.2)	1.01 (0.78-1.30, p=0.964)	1.03 (0.76-1.38, p=0.868)
Radiation	None	138 (46.6)	-	-
	WBRT	91 (30.7)	0.75 (0.57-0.99, p=0.039)	0.74 (0.54-1.01, p=0.061)
	SRS	67 (22.6)	0.49 (0.36-0.68, p<0.001)	0.50 (0.34-0.73, p<0.001)
Surgery	None	77 (26.0)	-	-
	Yes	219 (74.0)	0.53 (0.40-0.70, p<0.001)	0.44 (0.31-0.61, p<0.001)

Abbreviations: HR: Hazard Ratio; p: p-value; SRS: Stereotactic Radiosurgery; WBRT: Whole Brain Radiation Therapy.

Supplementary Table S8. Reduced Model of the Cox Proportional Hazards Analysis with Treatment as One Variable Stratified by Education and Grade.

This analysis evaluates survival with treatment modeled as a single variable and stratification by education and tumor grade. Multiple treatment groups were significantly associated with poorer survival relative to the SRS+systemic reference. Older age remained an independent predictor of worse outcomes. No significant associations were found for race, facility type, histology, or diagnosis year in the adjusted model.

**Variable**	**Group**	**N (%)**	**HR (Univariable)**	**HR (Multivariable)**
Age group	40-64 years	123 (41.6)	-	-
	65-90 years	173 (58.4)	1.98 (1.54-2.55, p<0.001)	1.54 (1.16-2.03, p=0.003)
Race	White	259 (87.5)	-	-
	Non-white	37 (12.5)	0.81 (0.55-1.18, p=0.272)	0.71 (0.47-1.09, p=0.118)
Facility type	Academic	90 (30.4)	-	-
	Nonacademic	206 (69.6)	1.09 (0.84-1.41, p=0.530)	1.04 (0.79-1.38, p=0.759)
Histology type	Adenocarcinomas	278 (93.9)	-	-
	Non-adenocarcinomas	18 (6.1)	1.51 (0.93-2.45, p=0.092)	0.87 (0.48-1.54, p=0.625)
Year	2010-2015	186 (62.8)	-	-
	2016-2020	110 (37.2)	1.01 (0.78-1.30, p=0.964)	1.28 (0.97-1.70, p=0.083)
Treatment	SRS+Sys	49 (16.6)	-	-
	WBRT+Sys	51 (17.2)	1.67 (1.07-2.61, p=0.023)	1.98 (1.22-3.22, p=0.005)
	Sys	55 (18.6)	1.68 (1.09-2.59, p=0.019)	2.22 (1.36-3.60, p=0.001)
	SRS	18 (6.1)	3.79 (2.11-6.81, p<0.001)	4.15 (2.20-7.85, p<0.001)
	WBRT	40 (13.5)	2.72 (1.71-4.34, p<0.001)	3.71 (2.20-6.26, p<0.001)
	None	83 (28.0)	4.51 (3.01-6.75, p<0.001)	6.32 (3.97-10.06, p<0.001)

Abbreviations: HR: Hazard Ratio; p: p-value; SRS: Stereotactic Radiosurgery; SRS+Sys: Stereotactic Radiosurgery + systemic therapy; Sys: Systemic therapy; WBRT: Whole Brain Radiation Therapy; WBRT+Sys: Whole Brain Radiation Therapy + systemic therapy.

Supplementary Table S9. Cox Proportional Hazards Full Model 1, Stratified by Sex, Education, Tumor Grade, Histology, and Systemic Therapy.

This table presents univariable and multivariable hazard ratios (HR) for overall survival among patients, adjusting for a range of demographic, socioeconomic, clinical, and treatment-related factors. Stratification was applied for sex, education level (no high school), tumor grade, cancer type, and receipt of systemic therapy. Age ≥65 years was independently associated with worse survival (HR 1.60, p=0.034). Public insurance was associated with worse univariable survival (HR 1.72), but not significant after adjustment. No significant differences were observed by race, ethnicity, region, income, or year of diagnosis. Hazard ratios are shown with 95% confidence intervals and p-values for both univariable and adjusted models.

**Group**	**Variable**	**n (%)**	**HR (Univariable)**	**HR (Multivariable)**
Age group	40-64 years	123 (41.6)	-	-
	65-90 years	173 (58.4)	1.98 (1.54-2.55, p<0.001)	1.60 (1.04-2.48, p=0.034)
Race	White	259 (87.5)	-	-
	Non-white	37 (12.5)	0.81 (0.55-1.18, p=0.272)	0.63 (0.38-1.03, p=0.065)
Ethnicity	Non-Hispanic	268 (90.5)	-	-
	Hispanic	28 (9.5)	0.99 (0.65-1.49, p=0.945)	0.95 (0.55-1.64, p=0.856)
Facility type	Academic	90 (30.4)	-	-
	Nonacademic	206 (69.6)	1.09 (0.84-1.41, p=0.530)	1.09 (0.76-1.57, p=0.630)
Median income	<$74,063	182 (61.5)	-	-
	≥$74,063	114 (38.5)	1.10 (0.86-1.41, p=0.453)	1.09 (0.67-1.77, p=0.736)
Insurance type	Private	90 (30.4)	-	-
	Public	186 (62.8)	1.72 (1.30-2.26, p<0.001)	0.94 (0.60-1.48, p=0.796)
	Uninsured	20 (6.8)	0.71 (0.41-1.24, p=0.234)	0.64 (0.32-1.28, p=0.209)
Region	Metro	232 (78.4)	-	-
	Non-metro	64 (21.6)	0.95 (0.71-1.28, p=0.748)	1.15 (0.76-1.74, p=0.507)
Charlson-Deyo index	0	220 (74.3)	-	-
	1	43 (14.5)	1.32 (0.94-1.86, p=0.111)	1.65 (1.07-2.54, p=0.023)
	2-3	33 (11.1)	1.54 (1.04-2.26, p=0.029)	1.45 (0.88-2.39, p=0.141)

Tumor size	<40 mm	58 (19.6)	-	-
	40-70 mm	151 (51.0)	1.10 (0.79-1.52, p=0.585)	1.11 (0.74-1.68, p=0.613)
	>70 mm	87 (29.4)	1.12 (0.78-1.60, p=0.542)	1.37 (0.85-2.21, p=0.193)
Histology type	Adenocarcinomas	278 (93.9)	-	-
	Non-adenocarcinomas	18 (6.1)	1.51 (0.93-2.45, p=0.092)	0.89 (0.44-1.83, p=0.753)
Radiation	None	138 (46.6)	-	-
	WBRT	91 (30.7)	0.75 (0.57-0.99, p=0.039)	0.63 (0.43-0.93, p=0.019)
	SRS	67 (22.6)	0.49 (0.36-0.68, p<0.001)	0.37 (0.23-0.58, p<0.001)
Surgery	None	77 (26.0)	-	-
	Yes	219 (74.0)	0.53 (0.40-0.70, p<0.001)	0.40 (0.27-0.60, p<0.001)
Year	2010-2015	186 (62.8)	-	-
	2016-2020	110 (37.2)	1.01 (0.78-1.30, p=0.964)	1.05 0.74-1.50, p=0.775)

Abbreviations: HR: Hazard Ratio; p: p-value; SRS: Stereotactic Radiosurgery; WBRT: Whole Brain Radiation Therapy; Metro: Metropolitan; Non-metro: Non-metropolitan; Charlson-Deyo index: Comorbidity scoring index used for risk adjustment.

Supplementary Table S10. Cox Proportional Hazards Full Model 2, Stratified by Sex, Education Level, Tumor Grade, Cancer Type, and Treatment Summary.

This table summarizes the univariable and multivariable hazard ratios (HR) for overall survival, adjusting for demographic, clinical, and socioeconomic variables. Age ≥65 years was independently associated with worse survival (HR 1.88, p=0.019). No demographic, insurance, or socioeconomic variables showed significant associations with survival in the adjusted model. Charlson-Deyo score, tumor size, histology, and year of diagnosis were not independently associated with survival after adjustment. The treatment summary variable was handled through stratification and not directly reported in the HR. Hazard ratios are presented with 95% confidence intervals and corresponding p-values from both univariable and multivariable Cox models.

Group	Variable	n (%)	HR (Univariable)	HR (Multivariable)
Age group	40-64 years	123 (41.6)	-	-
	65-90 years	173 (58.4)	1.98 (1.54-2.55, p<0.001)	1.88 (1.11-3.20, p=0.019)
Race	White	259 (87.5)	-	-
	Non-white	37 (12.5)	0.81 (0.55-1.18, p=0.272)	0.63 (0.36-1.11, p=0.110)
Ethnicity	Non-Hispanic	268 (90.5)	-	-
	Hispanic	28 (9.5)	0.99 (0.65-1.49, p=0.945)	0.89 (0.49-1.64, p=0.720)
Facility type	Academic	90 (30.4)	-	-
	Nonacademic	206 (69.6)	1.09 (0.84-1.41, p=0.530)	0.91 (0.60-1.38, p=0.660)
Median income	<$74,063	182 (61.5)	-	-
	≥$74,063	114 (38.5)	1.10 (0.86-1.41, p=0.453)	1.03 (0.57-1.84, p=0.933)
Insurance type	Private	90 (30.4)	-	-
	Public	186 (62.8)	1.72 (1.30-2.26, p<0.001)	0.78 (0.46-1.34, p=0.366)
	Uninsured	20 (6.8)	0.71 (0.41-1.24, p=0.234)	0.87 (0.41-1.86, p=0.724)
Region	Metro	232 (78.4)	-	-
	Non-metro	64 (21.6)	0.95 (0.71-1.28, p=0.748)	1.41 (0.85-2.34, p=0.177)
Charlson-Deyo index	0	220 (74.3)	-	-
	1	43 (14.5)	1.32 (0.94-1.86, p=0.111)	1.48 (0.88-2.47, p=0.137)

	2-3	33 (11.1)	1.54 (1.04-2.26, p=0.029)	1.12 (0.60-2.09, p=0.712)
Tumor size	<40 mm	58 (19.6)	-	-
	40-70 mm	151 (51.0)	1.10 (0.79-1.52, p=0.585)	1.12 (0.68-1.82, p=0.660)
	>70 mm	87 (29.4)	1.12 (0.78-1.60, p=0.542)	1.39 (0.80-2.44, p=0.245)
Histology type	Adenocarcinomas	278 (93.9)	-	-
	Non-adenocarcinomas	18 (6.1)	1.51 (0.93-2.45, p=0.092)	0.67 (0.30-1.50, p=0.330)
Year	2010-2015	186 (62.8)	-	-
	2016-2020	110 (37.2)	1.01 (0.78-1.30, p=0.964)	1.12 0.76-1.66, p=0.554)

Abbreviations: HR: Hazard Ratio; p: p-value; Metro: Metropolitan; Non-metro: Non-metropolitan; Charlson-Deyo index: Comorbidity scoring index used for risk adjustment.

Supplementary Table S11. Accelerated Failure Time (AFT) Model 1: Full Model with Treatment as Three Separate Variables.

Time ratios (TRs) and 95% confidence intervals (CIs) were estimated from a log-linear AFT model evaluating factors associated with overall survival. Treatment was modeled as three distinct variables: radiation modality (WBRT or SRS), systemic therapy, and surgical resection. TR >1 indicates longer survival; TR <1 indicates shorter survival relative to the reference group.

This AFT model evaluates the effect of clinical, demographic, and treatment-related variables on overall survival. Treatment modalities [SRS (TR = 2.24), systemic therapy (TR = 2.25), and surgery (TR = 3.38)] were associated with prolonged survival (all $p < 0.001$). Whole-brain radiotherapy (TR = 1.49, $p = 0.025$) also conferred a survival benefit. Patients with lower educational attainment (<9.1% high school education) and those with public insurance had significantly shorter survival (TR = 0.60, $p = 0.008$ and TR = 0.66, $p = 0.046$, respectively). Conversely, non-white race was associated with longer survival (TR = 1.70, $p = 0.027$). Other factors including age, income, comorbidity burden, tumor size, and year of diagnosis were not significant in the multivariable model.

**Variable**	**Time Ratio (TR)**	**Std. Error (SE)**	**95% CI**	**P-Value**
Age 40–64 (ref)	—	—	—	—
Age 65–90	0.70	0.197	0.49–1.01	0.065
Male (ref)	—	—	—	—
Female	0.94	0.150	0.70–1.27	0.666
White (ref)	—	—	—	—
Non-white	1.70	0.240	1.06–2.73	0.027
Non-Hispanic (ref)	—	—	—	—
Hispanic	1.14	0.252	0.70–1.85	0.610
Academic facility (ref)	—	—	—	—
Nonacademic	0.79	0.172	0.56–1.11	0.182
Income <$74,063 (ref)	—	—	—	—
Income ≥$74,063	1.46	0.217	0.95–2.25	0.083
Private insurance (ref)	—	—	—	—
Public insurance	0.66	0.212	0.45–0.98	0.046
Uninsured	1.36	0.339	0.70–2.62	0.358
≥9.1% HS education (ref)	—	—	—	—
<9.1% HS education	0.60	0.191	0.42–0.84	0.008
Metro (ref)	—	—	—	—
Non-metro	1.19	0.199	0.81–1.75	0.380

Charlson-Deyo = 0 (ref)	—	—	—	—
Charlson-Deyo = 1	0.72	0.207	0.48–1.08	0.106
Charlson-Deyo = 2–3	0.84	0.239	0.53–1.34	0.463
Tumor size <40 mm (ref)	—	—	—	—
Tumor size 40–70 mm	0.96	0.200	0.65–1.43	0.844
Tumor size >70 mm	0.82	0.216	0.54–1.25	0.356
Grade I (ref)	—	—	—	—
Grade II	1.98	0.341	1.01–3.87	0.045
Grade III	1.14	0.344	0.58–2.22	0.710
Grade IV	0.68	0.419	0.30–1.52	0.358
Colon (ref)	—	—	—	—
Rectum	1.01	0.207	0.66–1.54	0.977
Adenocarcinoma (ref)	—	—	—	—
Non-adenocarcinoma	1.36	0.329	0.72–2.56	0.352
No radiation (ref)	—	—	—	—
WBRT	1.49	0.178	1.05–2.10	0.025
SRS	2.24	0.212	1.49–3.38	<0.001
No systemic therapy (ref)	—	—	—	—
Systemic therapy	2.25	0.171	1.63–3.09	<0.001
No surgery (ref)	—	—	—	—
Surgery	3.38	0.189	2.36–4.84	<0.001
Year 2010–2015 (ref)	—	—	—	—
Year 2016–2020	0.91	0.169	0.66–1.26	0.584

Abbreviations: TR: Time Ratio; SE: Standard Error; CI: Confidence Interval; p: p-value; HS: High School; SRS: Stereotactic Radiosurgery; WBRT: Whole Brain Radiation Therapy; Metro: Metropolitan; Non-metro: Non-metropolitan; Charlson-Deyo index: Comorbidity scoring index.

Supplementary Table S12. Accelerated Failure Time (AFT) Model 2: Full Model with Treatment as a Single Summary Variable.

Time ratios (TRs) and 95% confidence intervals (CIs) were estimated from a log-linear AFT model evaluating factors associated with overall survival. Treatment was modeled as a single summary variable incorporating combinations of radiation and systemic therapy. A TR >1 indicates longer survival; TR <1 indicates shorter survival relative to the reference group.

This AFT model explores demographic, clinical, and treatment-related predictors of overall survival. Treatment regimens were significantly associated with survival: patients receiving WBRT+systemic therapy (TR = 0.47, p = 0.010), systemic therapy only (TR = 0.38, p = 0.001), SRS (TR = 0.22, p < 0.001), and WBRT alone (TR = 0.25, p < 0.001) had significantly shorter survival compared to untreated patients, who had the lowest TR (0.15, p < 0.001), suggesting these were modeled inversely.

Lower educational attainment (<9.1% high school education; TR = 0.56, p = 0.006) and higher comorbidity (Charlson-Deyo = 1; TR = 0.59, p = 0.017) were independently associated with shorter survival. Conversely, non-white race was associated with longer survival (TR = 1.67, p = 0.048). Other variables including age (TR = 0.66, p = 0.050), insurance type, income, tumor size, grade, and year of diagnosis were not statistically significant in this model.

Variable	Time Ratio (TR)	Std. Error (SE)	95% CI	p-value
Age 40–64 (ref)	—	—	—	—
Age 65–90	0.66	0.214	0.44–1.00	0.050
Male (ref)	—	—	—	—
Female	1.00	0.161	0.73–1.37	0.989
White (ref)	—	—	—	—
Non-white	1.67	0.259	1.00–2.79	0.048
Non-Hispanic (ref)	—	—	—	—
Hispanic	1.01	0.279	0.59–1.72	0.965
Academic facility (ref)	—	—	—	—
Nonacademic	1.03	0.180	0.72–1.48	0.869
Income <$74,063 (ref)	—	—	—	—
Income ≥$74,063	1.44	0.236	0.91–2.26	0.121
Private insurance (ref)	—	—	—	—
Public insurance	0.72	0.232	0.46–1.12	0.161
Uninsured	1.55	0.369	0.75–3.20	0.237
≥9.1% HS education (ref)	—	—	—	—
<9.1% HS education	0.56	0.212	0.37–0.84	0.006
Metro (ref)	—	—	—	—

Non-metro	1.11	0.219	0.72–1.70	0.639
Charlson-Deyo = 0 (ref)	—	—	—	—
Charlson-Deyo = 1	0.59	0.218	0.39–0.89	0.017
Charlson-Deyo = 2–3	0.73	0.260	0.44–1.23	0.234
Tumor size <40 mm (ref)	—	—	—	—
Tumor size 40–70 mm	1.07	0.220	0.70–1.63	0.743
Tumor size >70 mm	1.00	0.232	0.63–1.58	0.995
Grade I (ref)	—	—	—	—
Grade II	1.55	0.362	0.75–3.23	0.229
Grade III	1.05	0.371	0.50–2.18	0.900
Grade IV	0.72	0.453	0.30–1.72	0.474
Colon (ref)	—	—	—	—
Rectum	0.75	0.214	0.49–1.16	0.183
Adenocarcinoma (ref)	—	—	—	—
Non-adenocarcinoma	0.94	0.346	0.47–1.90	0.854
No treatment (ref)	—	—	—	—
WBRT+Systemic therapy	0.47	0.297	0.26–0.86	0.010
Systemic therapy only	0.38	0.291	0.21–0.70	0.001
SRS	0.22	0.393	0.10–0.48	0.000
WBRT	0.25	0.314	0.13–0.51	0.000
No treatment	0.15	0.272	0.09–0.26	0.000
Year 2010–2015 (ref)	—	—	—	—
Year 2016–2020	0.73	0.178	0.52–1.04	0.072

Abbreviations: TR: Time Ratio; SE: Standard Error; CI: Confidence Interval; p: p-value; HS: High School; SRS: Stereotactic Radiosurgery; WBRT: Whole Brain Radiation Therapy; Metro: Metropolitan; Non-metro: Non-metropolitan; Charlson-Deyo index: Comorbidity scoring index.

Supplementary Figure S1: OR Plot with Time Cutoff at 12 Months.

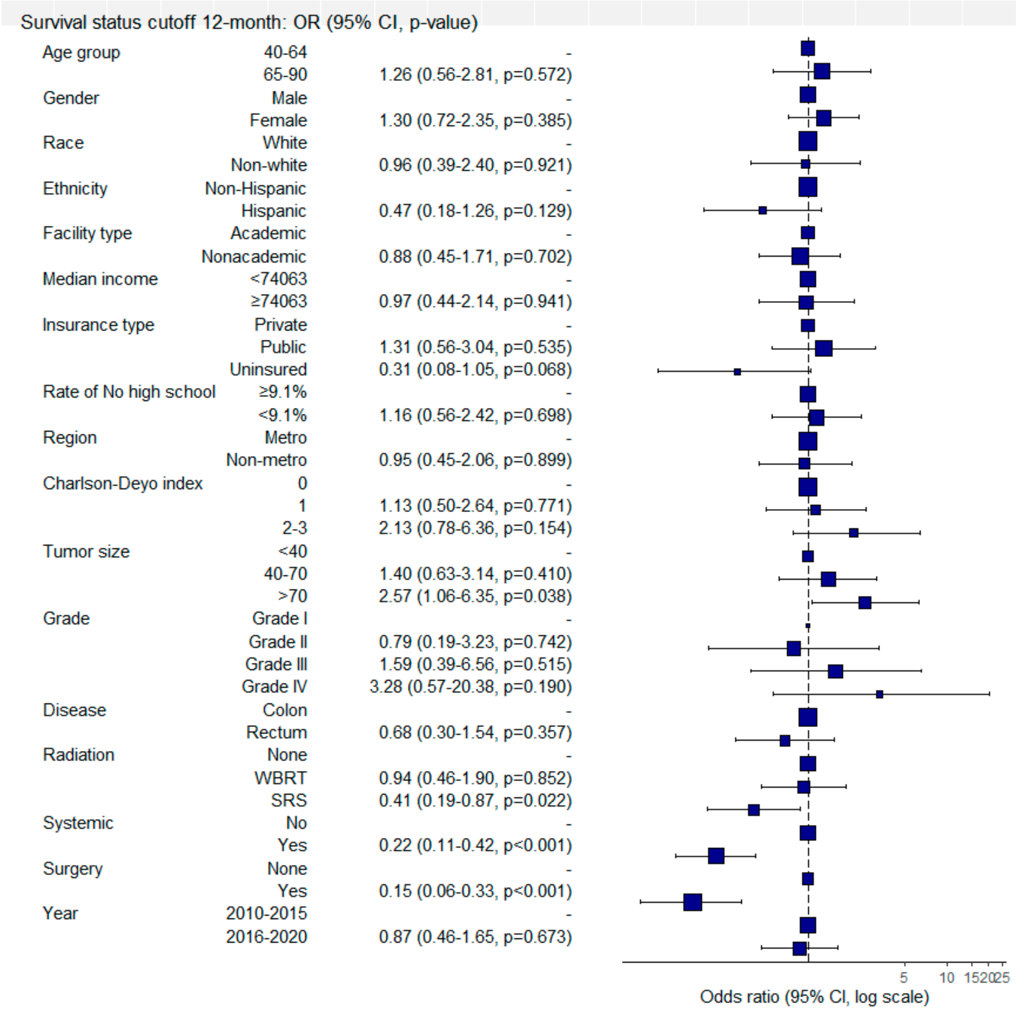

Abbreviations: OR: Odds Ratio; CI: Confidence Interval; p: p-value; SRS: Stereotactic Radiosurgery; SRS+Sys: Stereotactic Radiosurgery + systemic therapy; Sys: Systemic therapy; WBRT: Whole Brain Radiation Therapy; WBRT+Sys: Whole Brain Radiation Therapy + systemic therapy; Charlson-Deyo index: Comorbidity scoring index.

Supplementary Figure S2: OR Plot with Time Cutoff at 18 Months.

Survival status cutoff 18-month: OR (95% CI, p-value)		
Age group	40-64	-
	65-90	1.24 (0.51-2.98, p=0.630)
Gender	Male	-
	Female	0.74 (0.39-1.42, p=0.371)
Race	White	-
	Non-white	0.68 (0.27-1.77, p=0.413)
Ethnicity	Non-Hispanic	-
	Hispanic	0.92 (0.31-3.16, p=0.894)
Facility type	Academic	-
	Nonacademic	1.05 (0.51-2.13, p=0.889)
Median income	<74063	-
	≥74063	1.06 (0.45-2.54, p=0.894)
Insurance type	Private	-
	Public	1.43 (0.58-3.52, p=0.435)
	Uninsured	0.60 (0.18-2.04, p=0.406)
Rate of No high school	≥9.1%	-
	<9.1%	1.39 (0.63-3.17, p=0.419)
Region	Metro	-
	Non-metro	1.04 (0.45-2.43, p=0.934)
Charlson-Deyo index	0	-
	1	1.17 (0.47-3.13, p=0.749)
	2-3	1.88 (0.65-6.15, p=0.268)
Tumor size	<40	-
	40-70	2.76 (1.20-6.45, p=0.017)
	>70	3.30 (1.34-8.40, p=0.010)
Grade	Grade I	-
	Grade II	0.35 (0.07-1.50, p=0.183)
	Grade III	0.67 (0.12-2.89, p=0.614)
	Grade IV	1.62 (0.21-13.00, p=0.638)
Disease	Colon	-
	Rectum	0.58 (0.24-1.41, p=0.224)
Radiation	None	-
	WBRT	0.58 (0.26-1.28, p=0.180)
	SRS	0.26 (0.12-0.59, p=0.001)
Systemic	No	-
	Yes	0.31 (0.15-0.63, p=0.002)
Surgery	None	-
	Yes	0.11 (0.04-0.28, p<0.001)
Year	2010-2015	-
	2016-2020	0.63 (0.32-1.27, p=0.198)

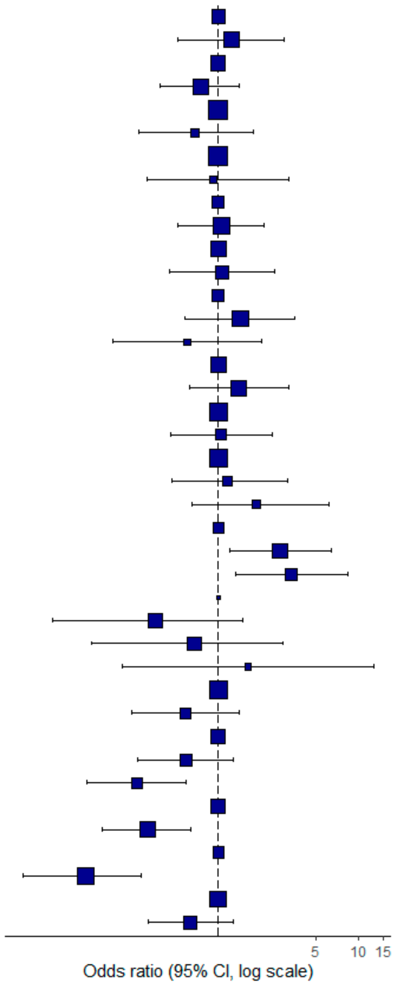

Abbreviations: OR: Odds Ratio; CI: Confidence Interval; p: p-value; SRS: Stereotactic Radiosurgery; WBRT: Whole Brain Radiation Therapy; Systemic: Systemic therapy (e.g., chemotherapy or immunotherapy)

Supplementary Figure S3: OR Plot with Time Cutoff at 24 Months.

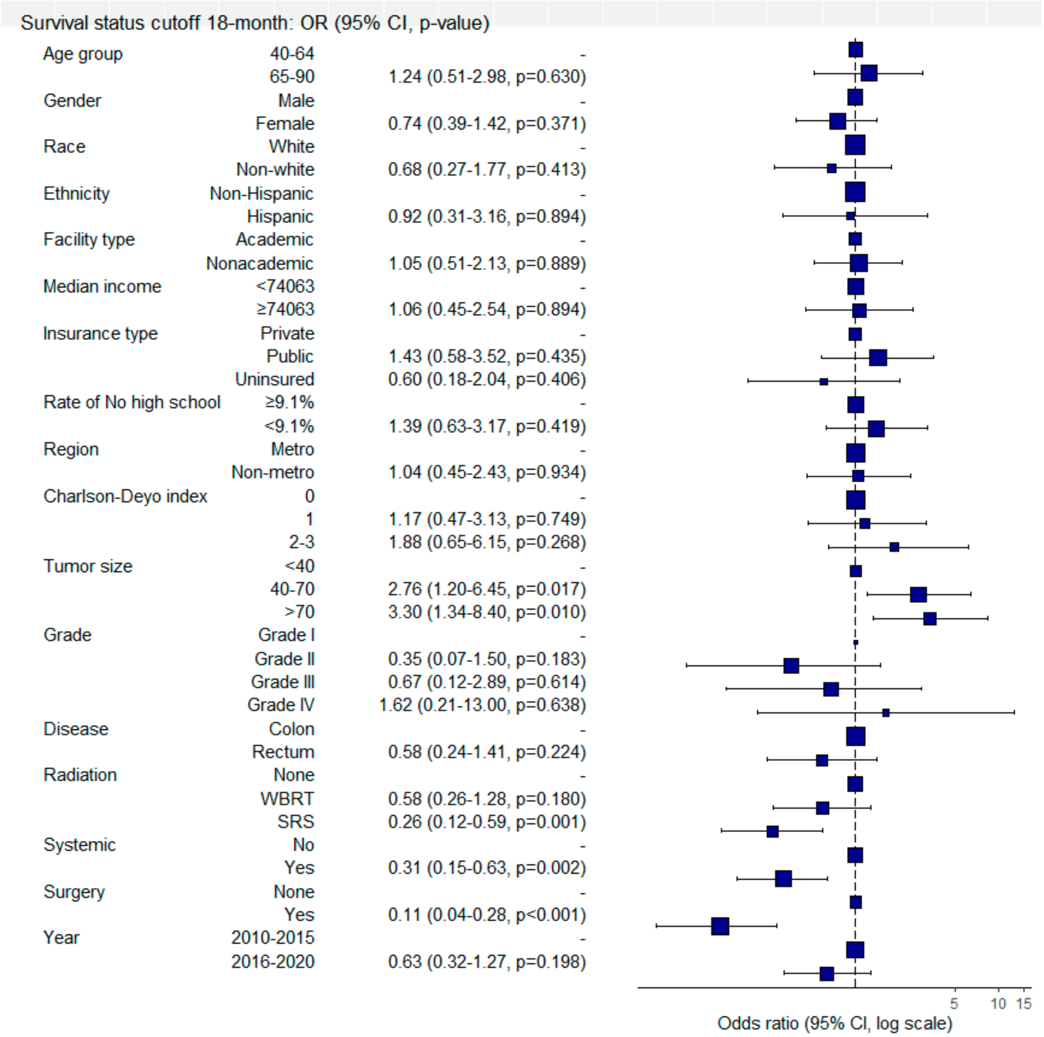

Abbreviations: OR: Odds Ratio; CI: Confidence Interval; p: p-value; SRS: Stereotactic Radiosurgery; WBRT: Whole Brain Radiation Therapy; Systemic: Systemic therapy (e.g., chemotherapy or immunotherapy)

Supplementary Figure S4: Assessment of the Proportional Hazards Assumption for Age Group.

The smoothed line is relatively flat and centered around zero, with confidence bands (dashed lines) mostly parallel and not diverging significantly. PH assumption likely holds for age group. No strong time-varying effect observed.

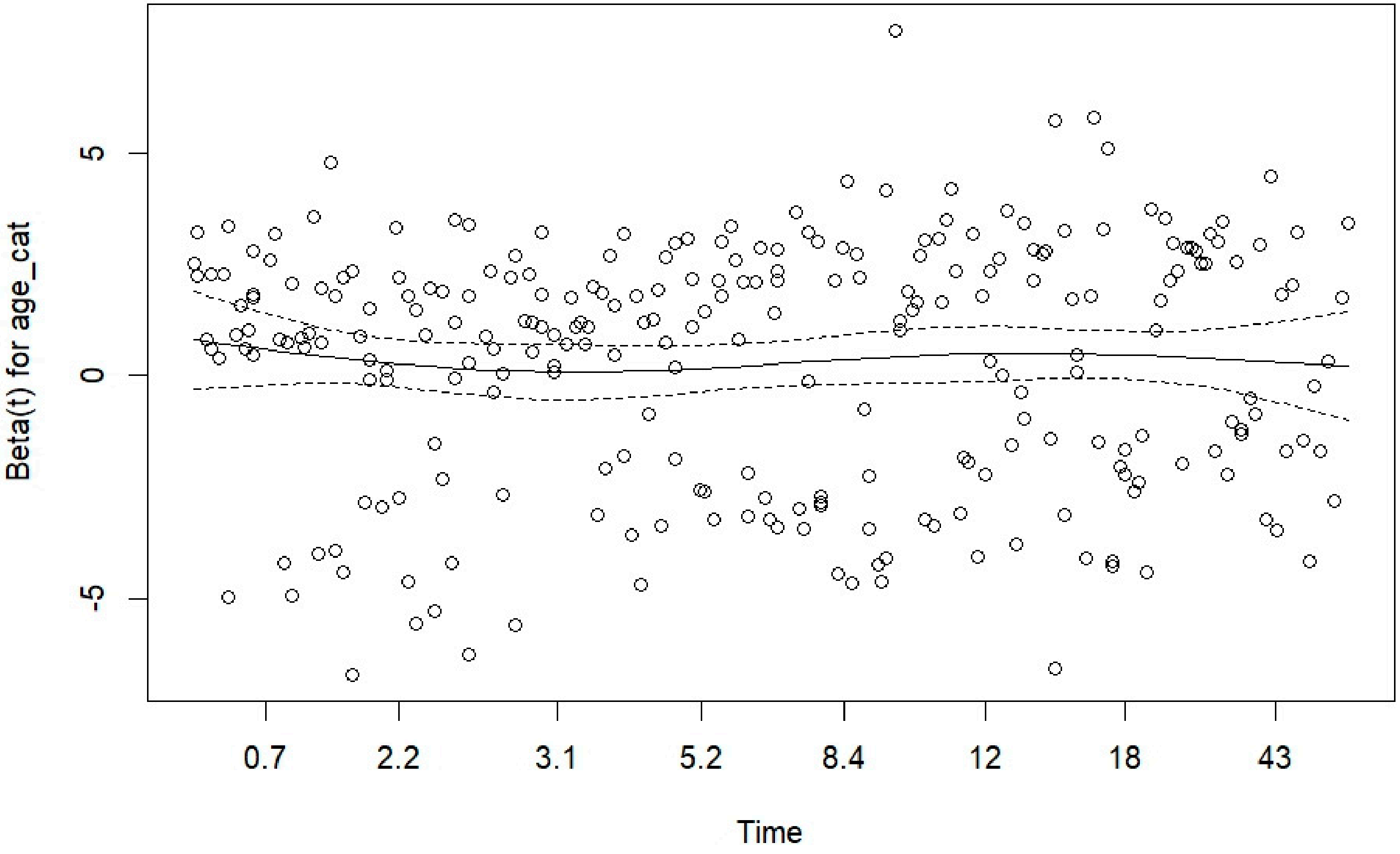

Supplementary Figure S5: Assessment of the Proportional Hazards Assumption for Charlson-Deyo Comorbidity Index

Large scatter, but the smoothed line stays relatively stable. Some widening of confidence intervals at extremes. Generally acceptable; though there is variance, no clear trend violating PH assumption.

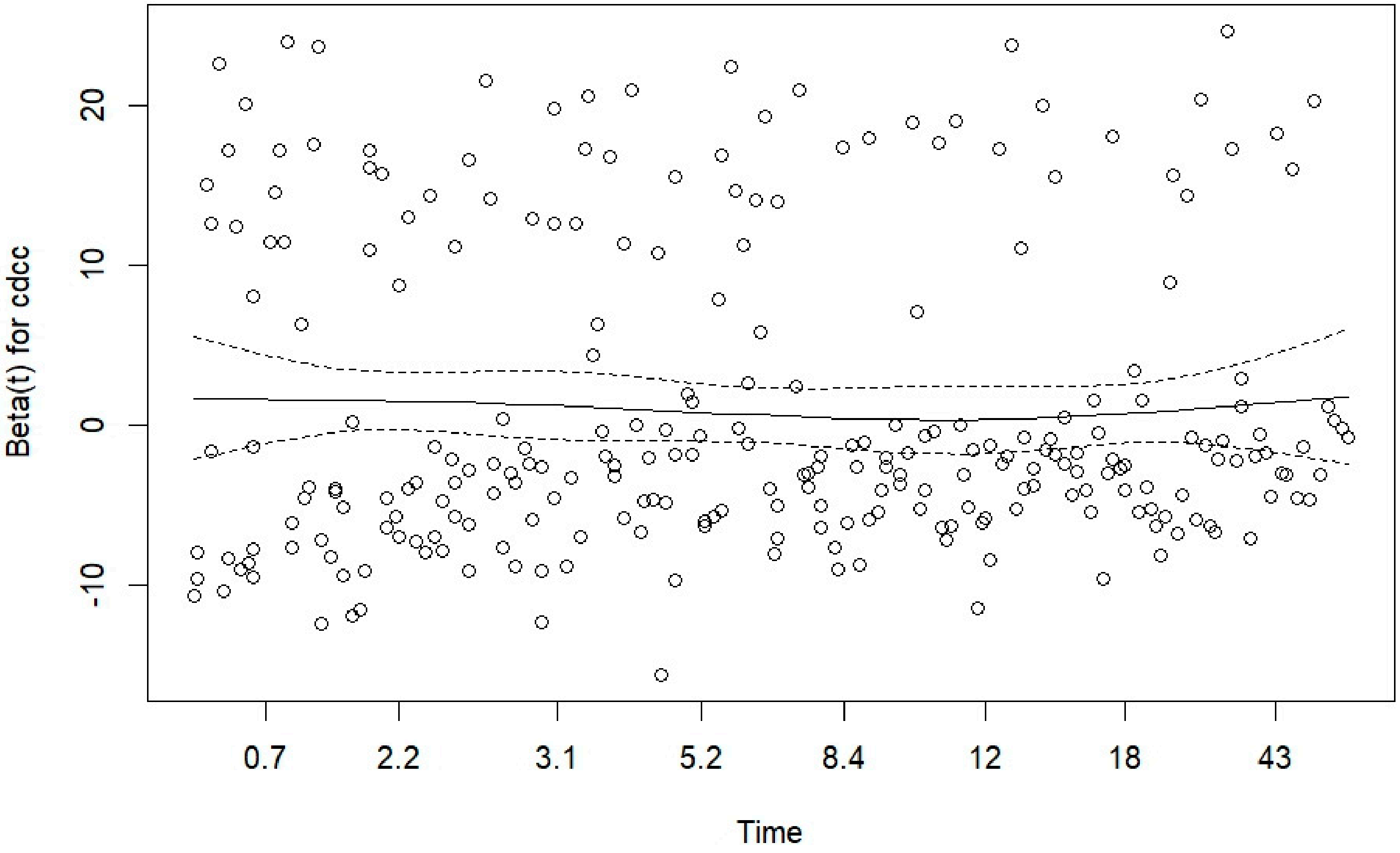

Supplementary Figure S6. Assessment of the Proportional Hazards Assumption for Ethnicity.

Flat smooth curve close to zero. Some high outliers, but no consistent trend over time. PH assumption likely valid. Outliers do not suggest a systemic time-dependent effect.

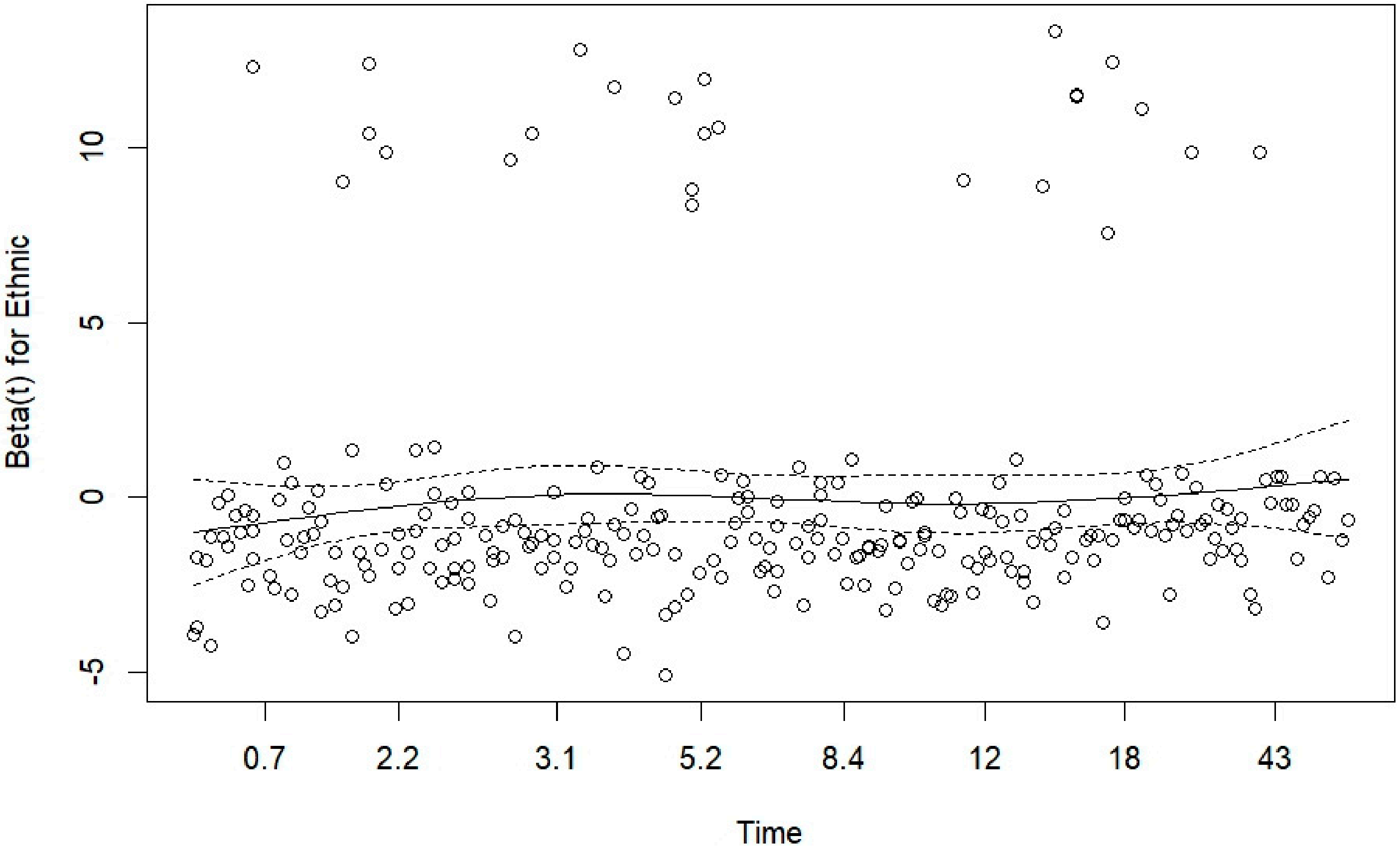

Supplementary Figure S7. Assessment of the Proportional Hazards Assumption for Histology Type.

Smoothed line is stable and close to zero, despite some vertical spread. PH assumption likely satisfied. No evidence of non-proportional hazards.

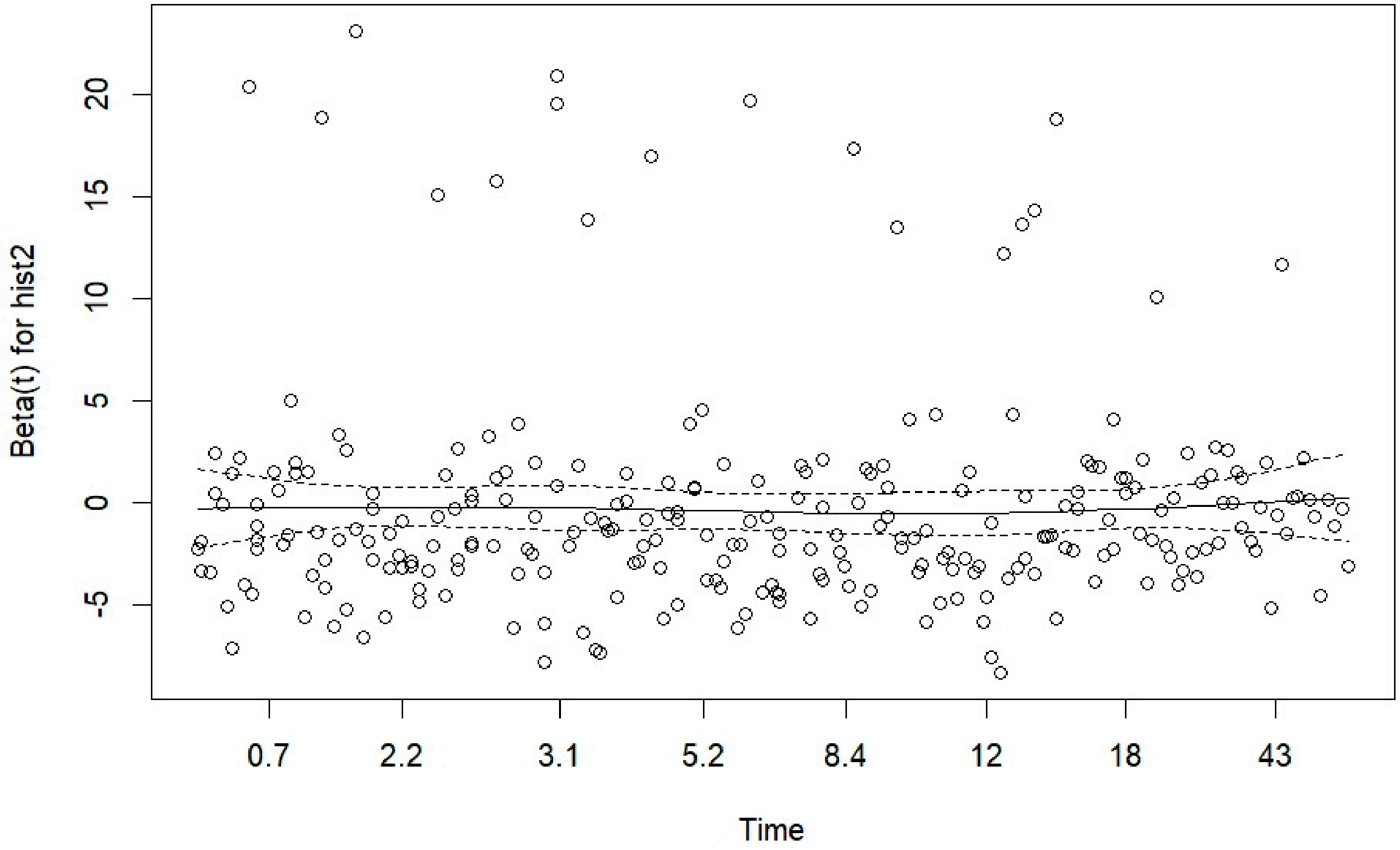

Supplementary Figure S8. Assessment of the Proportional Hazards Assumption for Insurance Type.

Slight upward trend in the smoothed line over time, and wider confidence intervals in the tail. Possible violation of PH assumption for insurance type. Consider stratifying by insurance or modeling it with time-varying coefficients.

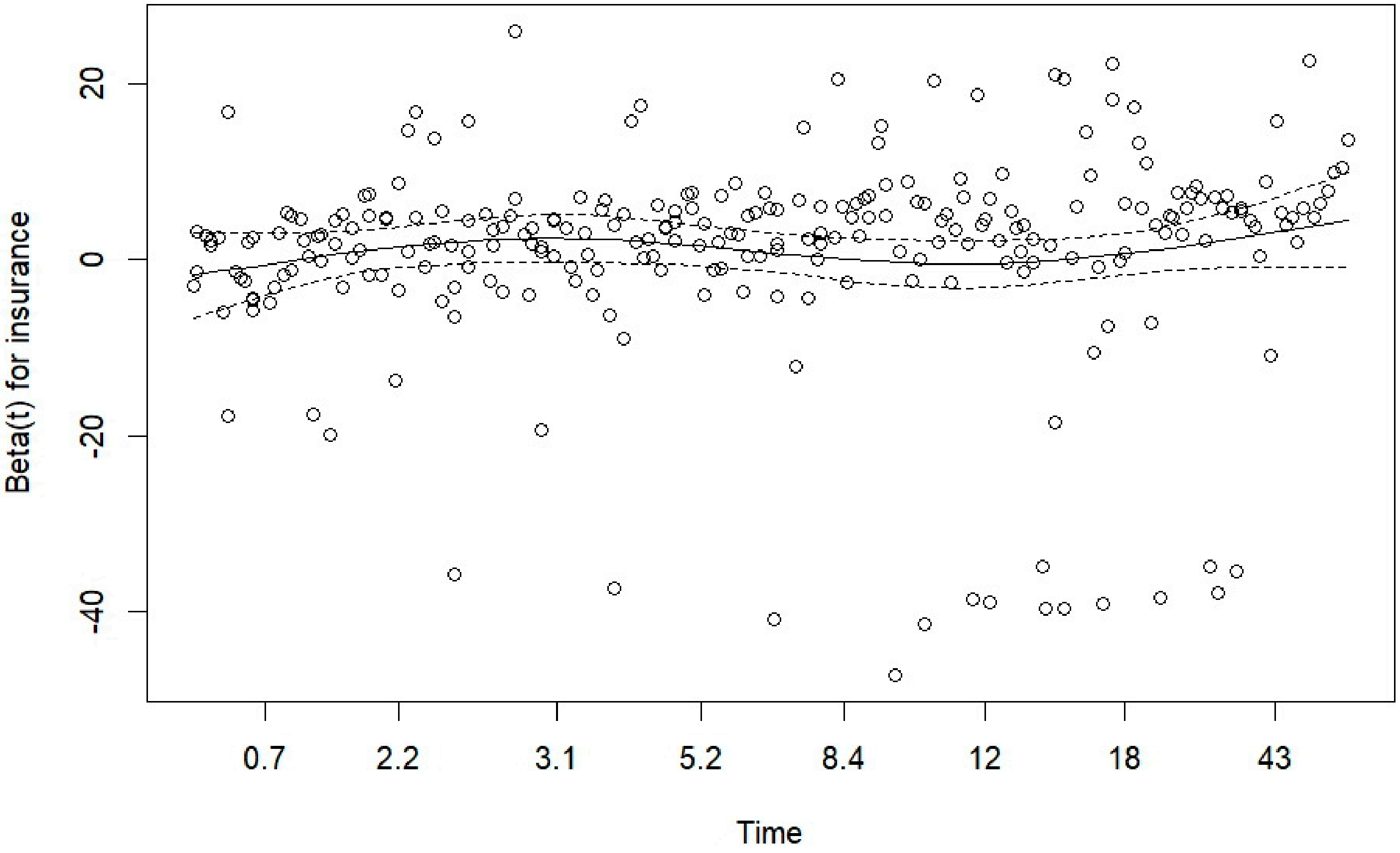

Supplementary Figure S9. Assessment of the Proportional Hazards Assumption for Race.

The smoothed line is slightly curved but stays relatively close to zero with parallel confidence bands. No major violation. The proportional hazards assumption appears reasonably met for race.

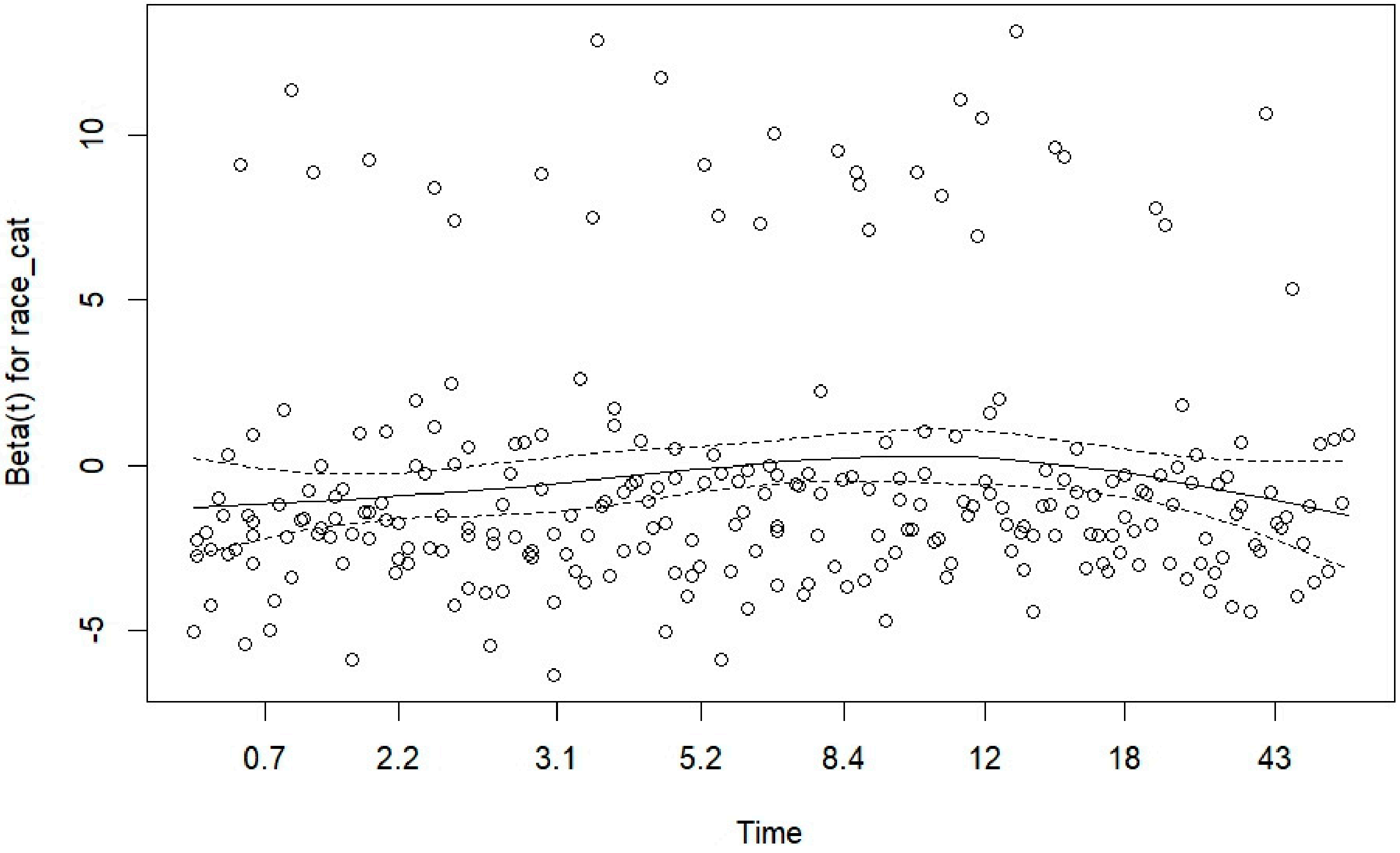

Supplementary Figure S10. Assessment of the Proportional Hazards Assumption for Radiation Therapy

There is a clear non-linear trend in the smoothed line, particularly an early dip and late rise, suggesting time-varying effects. Violation of PH assumption. Consider stratification or modeling radiation as a time-varying covariate.

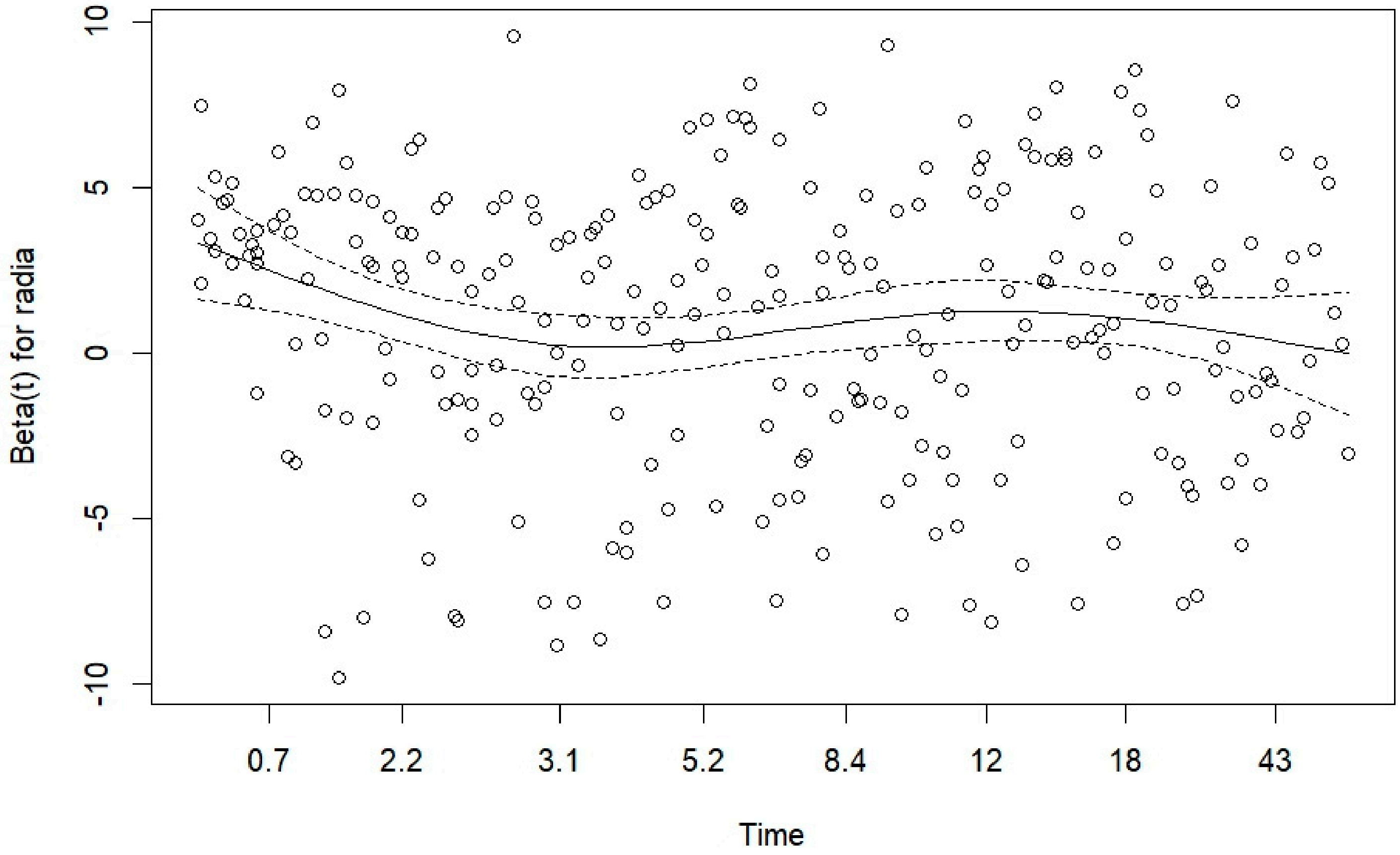

Supplementary Figure S11. Assessment of the Proportional Hazards Assumption for Surgery

The smoothed line is stable, remaining near zero. Confidence bands are relatively tight. PH assumption is likely satisfied.

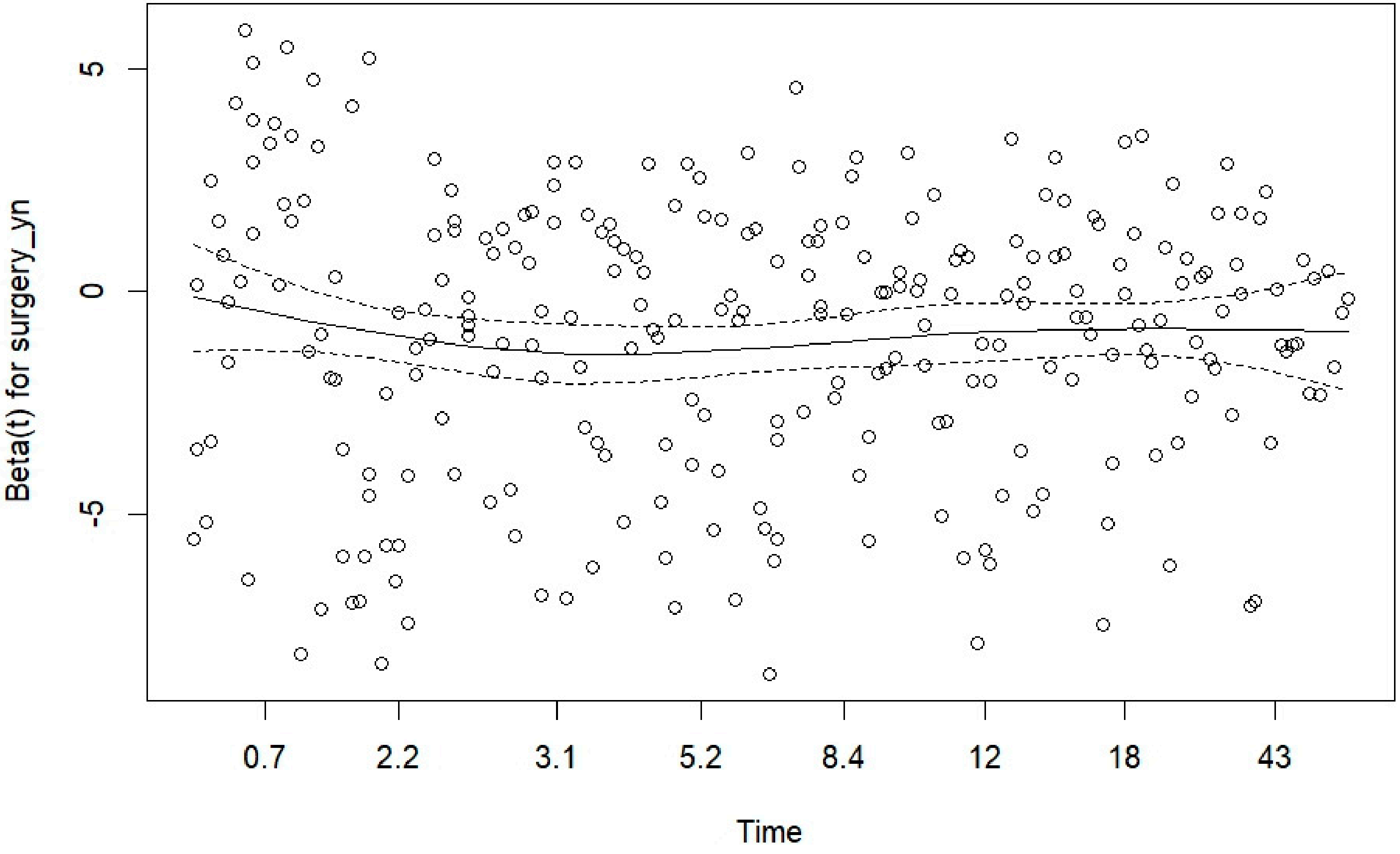

Supplementary Figure S12. Assessment of the Proportional Hazards Assumption for Systemic Therapy.

A moderate time-varying pattern is visible with upward slope and deviation from horizontal. Possible violation of PH assumption. May benefit from testing an interaction with time or stratification.

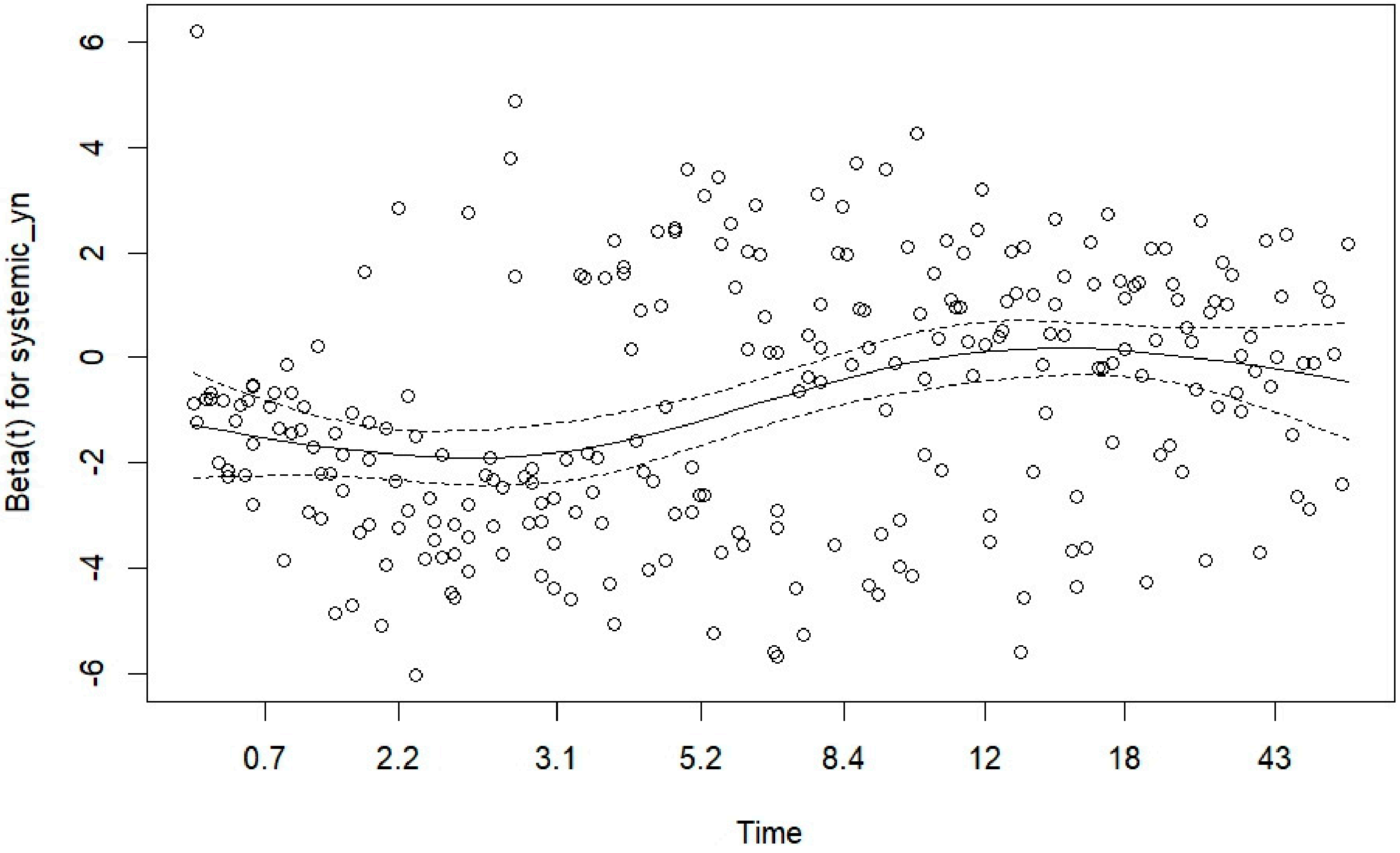

Supplementary Figure S13. Assessment of the Proportional Hazards Assumption for Composite Treatment Category

Clear downward trend in the smoothed line, with widening confidence intervals — indicating a non-constant effect over time. Strong violation of PH assumption. Treatment category should likely be handled with a time-varying effect model or stratification.

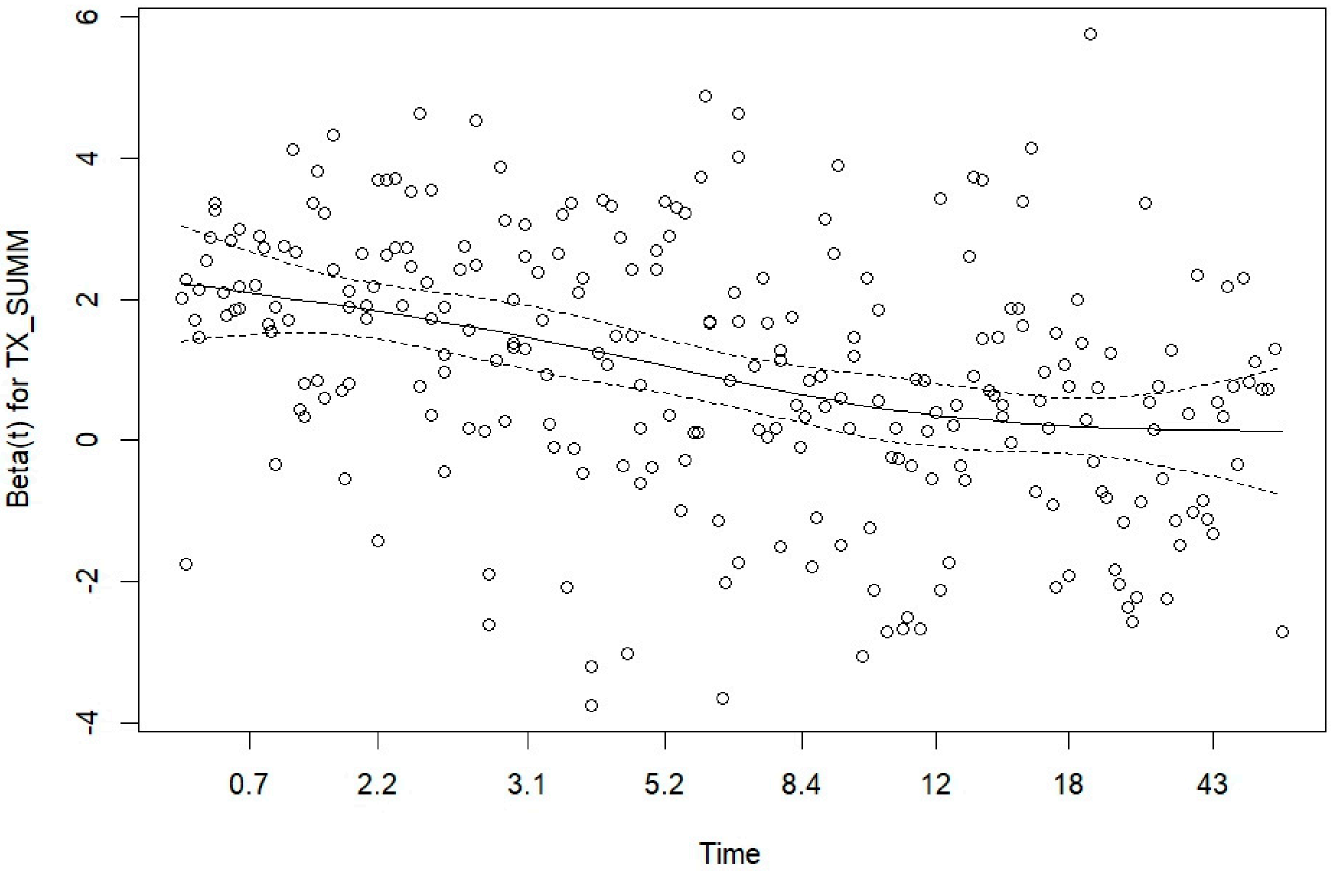

Supplementary Figure S14. Assessment of the Proportional Hazards Assumption for Tumor Size

The smoothed line shows a slight nonlinear curvature but overall stays close to 0. There is considerable vertical spread (residual variance), but the confidence bands remain fairly parallel and narrow across most of the time axis. No clear evidence of non-proportionality. The proportional hazards assumption appears to hold reasonably well for tumor size.

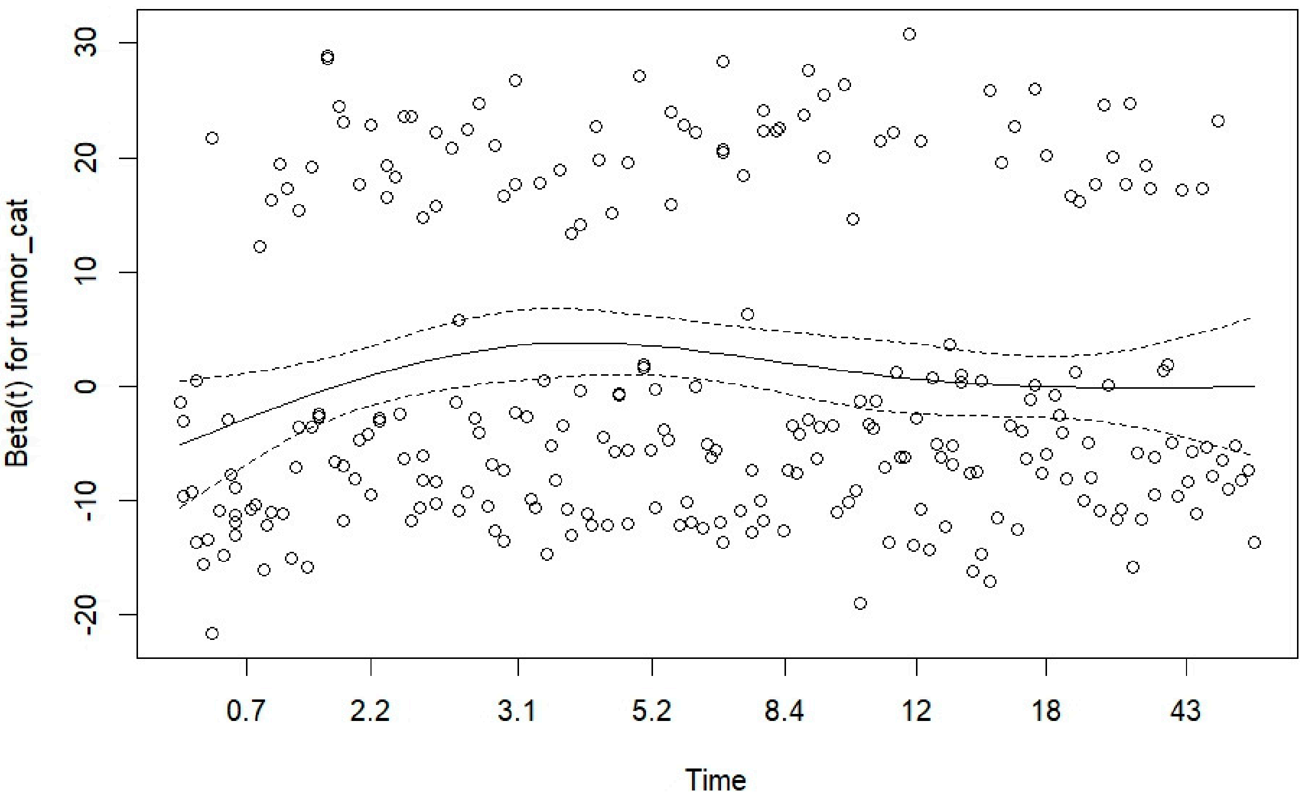

Supplementary Figure S15. Assessment of the Proportional Hazards Assumption for Year of Diagnosis

The smoothed line is nearly flat and centered around 0. The confidence bands are narrow and parallel across the time axis. Data points are symmetrically distributed with no discernible trend. Strong support for proportional hazards. Year of diagnosis satisfies the PH assumption.

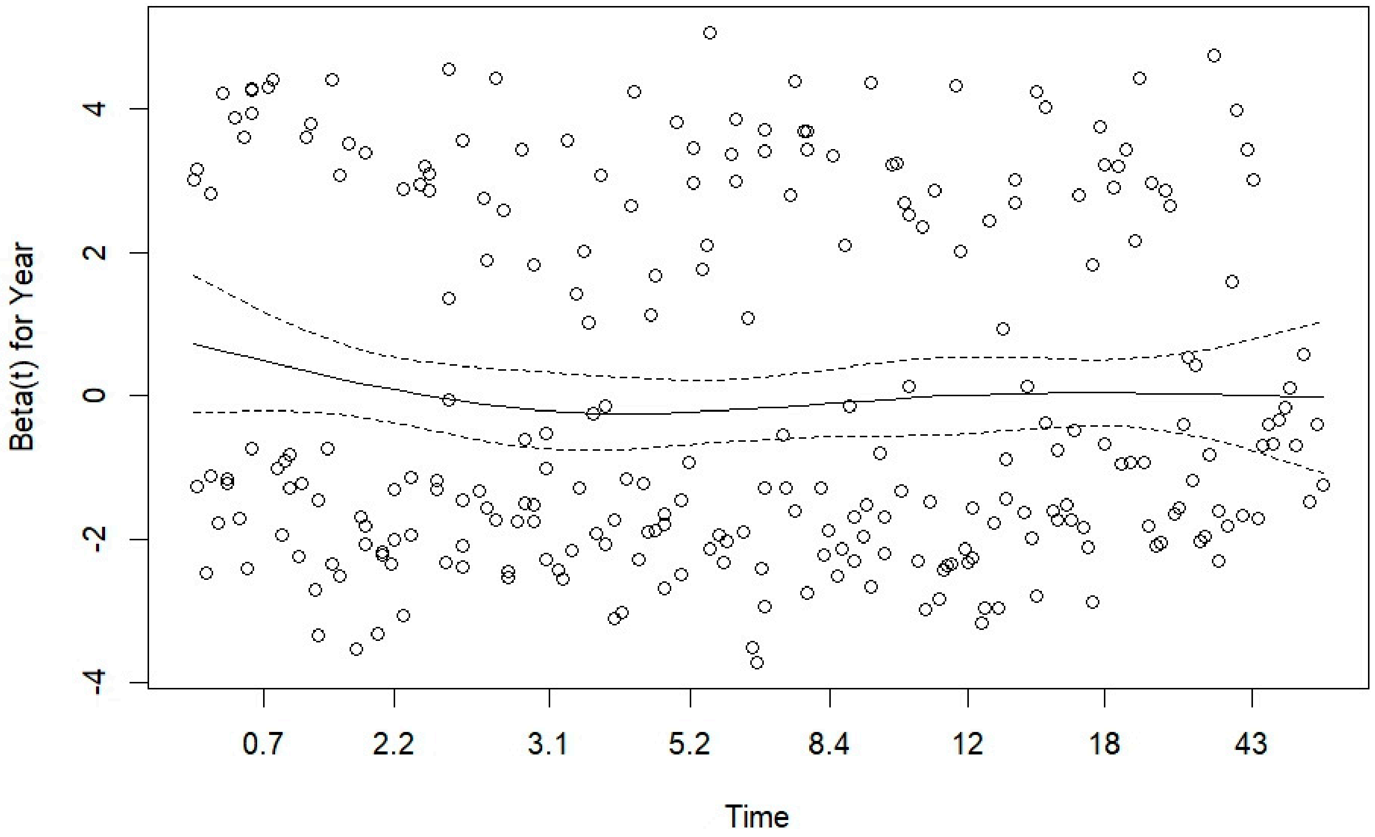

Supplement: Supplementary file 1 [file cancers-17-02531-s001.zip › cancers-3592974-supplementary.pdf]
